# Differential lactate and cholesterol synthetic activities in XY and XX Sertoli cells

**DOI:** 10.1038/srep41912

**Published:** 2017-02-02

**Authors:** Yurina Shishido, Takashi Baba, Tetsuya Sato, Yuichi Shima, Kanako Miyabayashi, Miki Inoue, Haruhiko Akiyama, Hiroshi Kimura, Yoshiakira Kanai, Yasuhiro Ishihara, Shogo Haraguchi, Akira Miyazaki, Damjana Rozman, Takeshi Yamazaki, Man-Ho Choi, Yasuyuki Ohkawa, Mikita Suyama, Ken-ichirou Morohashi

**Affiliations:** 1 Division of Molecular Life Sciences, Graduate School of Systems Life Science, Kyushu University, Maidashi 3-1-1, Higashi-ku, Fukuoka 812-8582, Japan; 2 Department of Molecular Biology, Graduate School of Medical Sciences, Kyushu University, Maidashi 3-1-1, Higashi-ku, Fukuoka 812-8582, Japan; 3 Division of Bioinformatics, Medical Institute of Bioregulation, Kyushu University, Maidashi 3-1-1, Higashi-ku, Fukuoka 812-8582, Japan; 4 AMED-CREST, Japan Agency for Medical Research and Development, Maidashi 3-1-1, Higashi-ku, Fukuoka 812-8582, Japan; 5 Department of Orthopaedics, Gifu University Graduate School of Medicine, Yanagito 1-1, Gifu, 501-1194, Japan; 6 Department of Biological Sciences, Graduate School of Bioscience and Biotechnology, Tokyo Institute of Technology, Yokohama, 226-8501, Japan; 7 Department of Veterinary Anatomy, The University of Tokyo, Yayoi 1-1-1, Bunkyo-ku, Tokyo 113-8657, Japan; 8 Graduate School of Integrated Arts and Sciences, Hiroshima University, Higashi-Hiroshima, 739-8521, Japan; 9 Department of Biochemistry, Showa University School of Medicine, Hatanodai 1-5-8, Shinagawa-ku, Tokyo 142-8555, Japan; 10 Centre for Functional Genomics and Bio-Chips, Institute of Biochemistry, Faculty of Medicine, University of Ljubljana, Zaloska 4, SI-1000 Ljubljana, Slovenia; 11 Molecular Recognition Research Center, Korea Institute of Science and Technology, Seoul, 02792, Korea; 12 Research Center for Transomics Medicine, Medical Institute of Bioregulation, Kyushu University, Maidashi 3-1-1, Higashi-ku, Fukuoka 812-8582, Japan

## Abstract

*SRY*, a sex-determining gene, induces testis development in chromosomally female (XX) individuals. However, mouse XX Sertoli cells carrying *Sry* (XX/*Sry* Sertoli cells) are incapable of fully supporting germ cell development, even when the karyotype of the germ cells is XY. While it has therefore been assumed that XX/*Sry* Sertoli cells are not functionally equivalent to XY Sertoli cells, it has remained unclear which specific functions are affected. To elucidate the functional difference, we compared the gene expression of XY and XX/*Sry* Sertoli cells. Lactate and cholesterol metabolisms, essential for nursing the developing germ cells, were down-regulated in XX/*Sry* cells, which appears to be caused at least in part by the differential expression of histone modification enzymes SMCX/SMCY (H3K4me3 demethylase) and UTX/UTY (H3K27me3 demethylase) encoded by the sex chromosomes. We suggest that down-regulation of lactate and cholesterol metabolism that may be due to altered epigenetic modification affects the nursing functions of XX/*Sry* Sertoli cells.

In mammals, the *SRY* gene (the sex determining region on the Y chromosome) has generally been thought to be sufficient for differentiation of the testes^1^,^2^,^3^. Indeed, an *Sry* transgene successfully induced testis development in XX fetuses; testicular cords were organized, Sertoli cells were differentiated within the cords, and Leydig cells were present in the interstitial space^4^. However, XX mice carrying an *Sry* transgene (XX/*Sry*) were found to be infertile^5^,^6^. Phenotypically, spermatogonial cells disappear from the testes soon after birth, and the presence of double X chromosomes has been suggested as a cause^7^. Moreover, since genes essential for spermatogenesis are localized on the Y chromosome^8^, XX germ cells are incapable of differentiating into matured male germ cells. The infertility of XX/*Sry* males has therefore been discussed from the viewpoint of a functional deficit of germ cells. It has, however, remained largely unclear whether XX/*Sry* Sertoli cells exhibit functions equivalent to XY Sertoli cells. Ishii *et al*.^6^ reported the interesting experimental observation that XY germ cells implanted into XX/*Sry* testes differentiated into round spermatids but rarely elongated spermatids. The authors concluded that the milieu established by XX/*Sry* Sertoli cells is insufficient for differentiation into elongated spermatids. However, the specific functions that have been affected in XX/*Sry* Sertoli cells still await clarification.

Since blood vessels are localized in the interstitial space outside the seminiferous tubules and Sertoli cells create a tight blood-testis barrier, nutrients and fuels for energy production cannot be supplied to germ cells via the blood. The Sertoli cells, often referred to as nursing cells, are responsible for the supply of energy and nutrients to the germ cells, with which they remain in close contact throughout the entire differentiation process^9^. Similar to nutrients, oxygen supply is restricted in the seminiferous tubule, and the testis has therefore been described as an oxygen-deprived organ^10^. In this unusual milieu, spermatocytes and mature sperms prefer lactate as fuel to produce ATP^11^. Sertoli cells produce lactate via glycolysis and then supply it to developing germ cells^12^,^13^.

Another fundamental material supplied to germ cells by Sertoli cells is cholesterol^14^. Sertoli cells are capable of synthesizing cholesterol by themselves, as well as absorbing it from high density lipoprotein (HDL)^15^,^16^. They also continuously phagocytose developing germ cells as another source of cholesterol^17^. Consequently, the quantity of intracellular cholesterol/cholesterol ester is regulated by the balance of synthesis, influx via the two above-mentioned routes, and efflux. It has been suggested that ATP-binding cassette transporter 1 (ABCA1) mediates cholesterol efflux from Sertoli cells, since disruption of *Abca1* gene led to defects in spermatogenesis together with unusual accumulation of lipids in the Sertoli cells^18^. In addition, gene knockout of retinoid X receptor β (Rxrb, Nr2b2)^19^ and double knockout of liver X receptor α/β (Lxrα, Nr1h3 and Lxrβ, Nr1h2)^20^ resulted in defects similar to *Abca1* gene knockout, possibly through down-regulation of *Abca1* gene transcription.

Sex chromosomes carry genes encoding histone modification enzymes such as SMCX (KDM5C)/SMXY (KDM5D) and UTX (KDM6A)/UTY. Both SMCX and SMCY mediate the demethylation of histone H3 trimethylated Lysine 4 (H3K4me3)^21^. UTX mediates the demethylation of histone 3 trimethylated Lysine 27 (H3K27me3), whereas such activity has not been found for UTY^22^,^23^. Evidence from multiple sources indicates that H3K4me3 accumulates predominantly around the transcription start sites of active genes, while H3K27me3 is distributed throughout gene bodies with inactive transcription^24^,^25^,^26^. The physiological function of *Utx* has been investigated using gene knockout mice^27^,^28^,^29^. Interestingly, in addition to affecting morphology, *Utx* was found to be required for sexually dimorphic deposits of H3K27me3^29^.

In the present study, we investigated the functional differences between XY and XX/*Sry* Sertoli cells by focusing on their role as nursing cells.

## Results

### Preparation of XY and XX/Sry Sertoli cells

**Figure 1:**
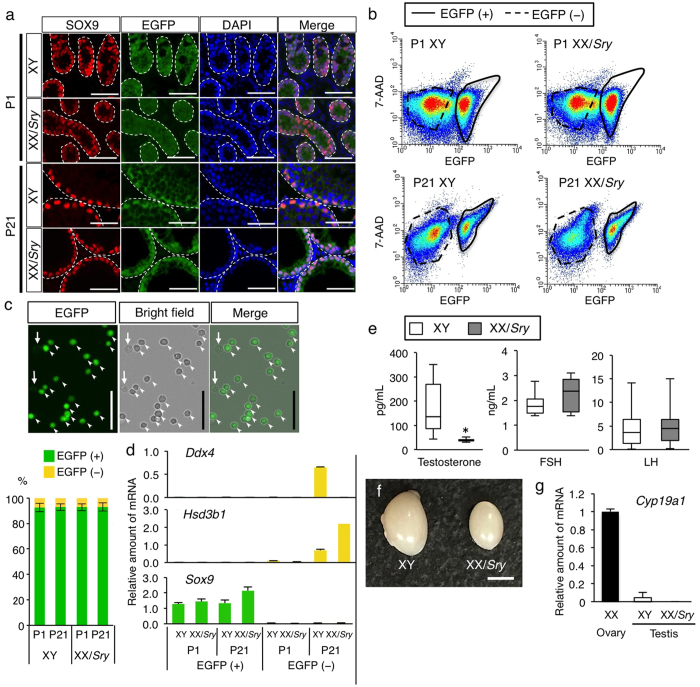
Preparation of XY and XX/*Sry* Sertoli cells. (**a)** Testes from XY and XX/*Sry* mice at P1 and P21 were immunostained with antibodies for SOX9 (red) and EGFP (green). Nuclei were stained with DAPI (blue). Merged images are shown in the right-hand panel. Seminiferous tubules are surrounded by white broken lines. Scale bars = 50 μm. (**b)** Testicular cells from XY and XX/*Sry* mice at P1 and P21 were fractioned by FACS. Fractions surrounded by solid lines were recovered as EGFP-positive cells, while fractions surrounded by broken lines were recovered as EGFP-negative cells. (**c)** Fluorescence and bright field images of the EGFP-positive cells from the P21 XY testes. The EGFP-positive (arrowheads) and EGFP-negative cells (arrows) were counted, and ratios of EGFP-positive (green) to EGFP-negative cells (yellow) are shown. (**d)** RNAs prepared from the EGFP-positive (green bars) and EGFP-negative (yellow bars) cells were used for qRT-PCR of *Ddx4* (germ cell marker), *Hsd3b1* (Leydig cell marker), and *Sox9* (Sertoli cell marker). The quantity of mRNA relative to *Actb* (encoding beta-actin) is indicated. (**e)** Concentration of testosterone, FSH, and LH were determined in XY and XX/*Sry* mice at P21. The blood samples for XY (n = 6) and XX/*Sry* mice (n = 6) were used for testosterone assays, while those for XY (n = 7) and XX/*Sry* mice (n = 6) were used for FSH and LH assays. *p < 0.05. (**f)** Whole view of XY and XX/*Sry* testes are shown. Scale bar = 2 mm. (**g)** The expression of *Cyp19* in XX ovary, and XY and XX/*Sry* testes was examined by qRT-PCR. The quantity of mRNA relative to *Actb* is indicated. Three biologically independent samples (n = 3) were used for the qRT-PCR studies in (**d)** and (**g)**.

To examine the contribution of sex chromosomes to gene expression in Sertoli cells, we used XY wild type and XX transgenic mice carrying the *Sry* transgene (XX/*Sry*). Sertoli cells from these mice were labeled with EGFP as described in ‘Materials and Methods’. As expected, all SOX9-positive (SRY-box containing gene 9) Sertoli cells were positive for EGFP in the testes of XX/*Sry* as well as XY wild type mice on postnatal days 1 and 21 (Fig. 1a). As reported previously^5^, germ cells had disappeared from the seminiferous tubules of the XX/*Sry* testes by P21. Whole testicular cells prepared from P1 and P21 testes were subjected to FACS. EGFP-positive and -negative cell fractions were recovered (Fig. 1b). Fluorescence microscopy indicated that more than 92% of the cells were EGFP-positive in all preparations (Fig. 1c).

Total RNAs were prepared from P1 and P21 EGFP-positive and -negative cells at and used for qRT-PCR analysis (Fig. 1d). As expected, *Sox9* mRNA was enriched in the EGFP-positive cell fractions, whereas a germ cell marker (homologue of a DEAD (Asp-Glu-Ala-Asp) family gene (*Ddx4, VASA*)), and a Leydig cell marker (*Hsd3b1 (Hydroxysteroid dehydrogenase Type 3b1*)) were enriched in the EGFP-negative cell fractions. Consistent with the disappearance of germ cells from XX/*Sry* testes, expression of *Ddx4* was much reduced in the EGFP-negative cell fraction of XX/*Sry* testes. Up-regulation of *Hsd3b1* in these cells might have resulted from an increased proportion of Leydig cells following the disappearance of the germ cells. Taken together, these marker gene expressions indicate that the EGFP-positive cell fractions prepared from the XY and XX/*Sry* testes comprised predominantly Sertoli cells.

Hormones necessary for reproductive activities were measured in XY and XX/*Sry* mice at P21. As shown in Fig. 1e, plasma testosterone was decreased in XX/*Sry* mice as compared to XY mice. Such significant alteration was not observed in the amounts of follicle stimulating hormone (FSH) or luteinizing hormone (LH). Consistent with the decreased testosterone concentration, the testicular size of XX/*Sry* mice was smaller than that of XY mice (Fig. 1f). Estradiol is a potent estrogen in females, but the concentration is too low to determine precisely in males. Therefore, we determined the expression level of the *Cyp19* gene, which is essential for the synthesis of estradiol. As shown in Fig. 1g, the expression of *Cyp19* in the XX ovary was higher than that in the XY testis. *Cyp19* gene expression was not observed in the XX/*Sry* testes, suggesting that estradiol could not be synthesized in the XX/*Sry* testis.

### Gene expression in XY and XX/Sry Sertoli cells

**Figure 2:**
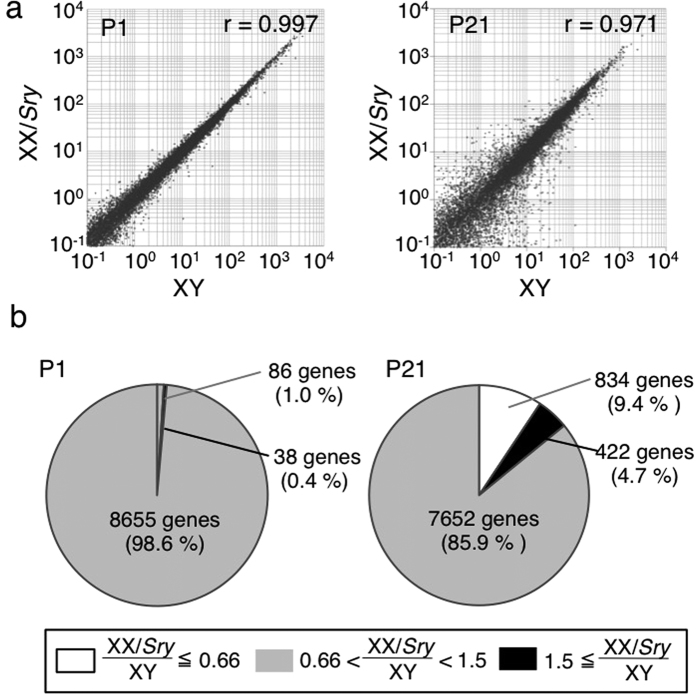
Gene expression in XY and XX/*Sry* Sertoli cells. (**a**) Comparison of gene expression levels (FPKM) in XY and XX/*Sry* Sertoli cells at P1 and P21. FPKM values of gene expression in XY Sertoli cells (*x*-axis) and XX/*Sry (y*-axis) cells are presented on a log scale; r: relative correlation. (**b**) Proportion of genes up- or down-regulated in XX/*Sry* Sertoli cells relative to XY Sertoli cells at P1 and P21.

The RNAs prepared from XY and XX/*Sry* Sertoli cells at P1 and P21 were sequenced. As summarized in Supplemental Table S1, approximately 30 million reads were obtained from every sample. More than 97% of reads were mapped to the reference genome, suggesting that the sequence data sets were of sufficient quality for further analyses. Gene expressions were compared between the two types of Sertoli cells at P1 and P21. Correlation coefficients between the cell types were 0.997 at P1 and 0.971 at P21, indicating that the gene expressions of the two types of Sertoli cells were very similar at P1 and differed slightly more at P21 (Fig. 2a). Consistent with this, 38 genes were up-regulated more than 1.5-fold and 86 genes down-regulated less than 1.5-fold in the P1 XX/*Sry* Sertoli cells, and 422 and 834 genes were respectively up- and down-regulated by the same margins in the P21 XX/*Sry* Sertoli cells (Fig. 2b).

Genes displaying differential expression are listed in Supplemental Tables S2–S5. *Sry* and *Xist* (inactive X-specific transcript) were treated as up-regulated genes in the P1 XX/*Sry* Sertoli cells (Supplemental Table S2). In the case of *Sry,* this was because the expression of the exogenous *Sry* gene is driven by *Hsp70.3* basal promoter. The increase of *Xist* suggests that X chromosome inactivation occurs even though the fate of cells carrying two X chromosomes is changed to male supporting Sertoli cells. Four Y-linked genes (*Smcy (Kdm5d), Ddx3y, Eif2s3y*, and *Uba1y*) were recorded as down-regulated in P1 XX/*Sry* Sertoli cells (Supplemental Table S3). This is consistent with the fact that the Y chromosome is absent from XX/*Sry* transgenic mice. Interestingly, the expression of genes for ribosomal protein (*Rpl36, Rps29, Rplp1*, and *Rps21*) and mitochondrial ribosomal protein (*Mrps12*) was decreased in the XX/*Sry* Sertoli cells.

Genes that were up- or down-regulated in P21 XX/*Sry* Sertoli cells are summarized in Supplemental Tables S4 and S5. We attempted to extract the biological events/pathways related to the listed genes by conducting GO and KEGG pathway analyses. Results are summarized in Supplemental Tables S6 and S7.

Genes up-regulated in P21 XX/*Sry* Sertoli cells were not distinguished by high fold enrichment and *P*-value (Supplemental Table S6), whereas this was not the case for down-regulated genes (Supplemental Table S7). Differences in the expression of genes with functions related to lactate metabolism and sterol/terpenoid metabolism were particularly noticeable and are discussed in the following sections.

### Lactate production decreased in XX/Sry Sertoli cells

**Figure 3:**
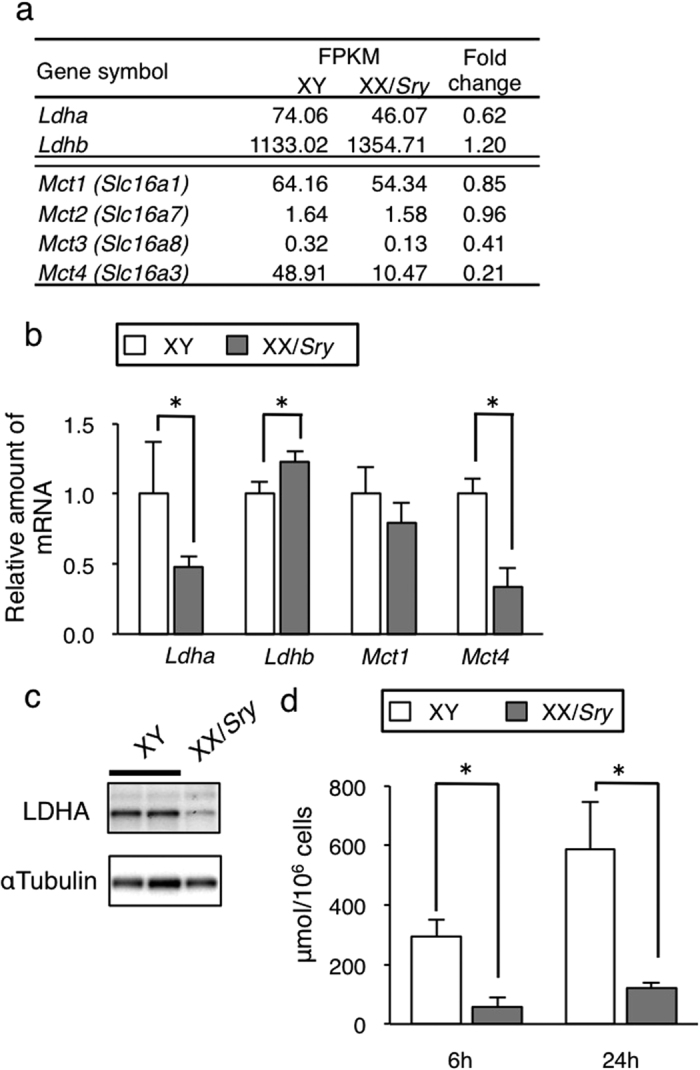
Lactate production decreased in XX/*Sry* Sertoli cells. (**a**) Expression levels of lactate dehydrogenase subunits (*Ldha* and *Ldhb*) and monocarboxylate transporters (*Mct1 (Slc16a1), Mct2 (Slc16a7), Mct3 (Slc16a8)*, and *Mct4 (Slc16a3)*) in XY and XX/*Sry* Sertoli cells at P21. (**b**) Expression of *Ldha, Ldhb, Mct1,* and *Mct4* in XY and XX/*Sry* Sertoli cells was validated by qRT-PCR analysis. The average values for XY Sertoli cells were normalized to 1.0. (**c**) Total cell lysates were prepared from XY and XX/*Sry* testes and then subjected to immunoblot analyses with antibodies for LDHA (upper) and α-tubuline (lower). (**d**) XY and XX/*Sry* Sertoli cells (5 × 10^5^) prepared from P21 testes were cultured. Quantities of lactate in the culture media were determined at 6 and 24 h after medium change. The study was performed three times with biologically independent Sertoli cell samples. Three biologically independent samples (n = 3) were used for the qRT-PCR studies in (**b** and **d)**. *p < 0.05.

It has been established that lactate supplied by Sertoli cells is utilized as an energy source by developing germ cells such as spermatocytes, spermatids, and spermatozoa^13^. Interestingly, genes related to ‘lactate dehydrogenase activity’ were among those down-regulated in P21 XX/*Sry* Sertoli cells. Lactate dehydrogenase (LDH) is a tetrameric enzyme of lactate dehydrogenase A (LDHA) and B (LDHB) subunits encoded by *Ldha* and *Ldhb*, respectively. Sequencing indicated that expression of *Ldha* was decreased in P21 XX/*Sry* Sertoli cells, while that of *Ldhb* was slightly increased (Fig. 3a). qRT-PCR analysis confirmed the sequence data (Fig. 3b). As expected, LDHA protein was decreased in P21 XX/*Sry* Sertoli cells (Fig. 3c).

LDH with four subunits of LDHA is known to preferentially mediate conversion from pyruvate to lactate. By contrast, LDH with four subunits of LDHB mediates conversion from lactate to pyruvate^30^,^31^. In addition to these, three distinct isoenzymes (one A/three B, two A/two B, and three A/one B) can be formed with LDHA and LDHB, and are thought to exhibit an intermediate level of activity^32^. Considering the decreased expression of *Ldha* and increased expression of *Ldhb* in the XX/*Sry* Sertoli cells, lactate production may be lower in these cells.

In addition to synthesis, the lactate transport activity of Sertoli cells should be considered. Monocarboxylate transporters encoded by *Mct1 (Slc16a1*), *Mct2 (Slc16a7*), *Mct3 (Slc16a8*), and *Mct4 (Slc16a3*) have been identified as lactate transporters. *Mct1* and *Mct4* were found to be expressed in the Sertoli cells, whereas *Mct2* and *Mct3* were mostly absent (Fig. 3a). Sequencing and qRT-PCR analysis indicated that expression of *Mct1* and *Mct4* was decreased slightly and strongly, respectively, in XX/*Sry* Sertoli cells (Fig. 3a,b).

MCT1 and MCT4 are responsible for the import and export of lactate, respectively^33^,^34^,^35^. Considering the down-regulated expression of both *Mct4* and lactate-synthesizing *Ldha*, the quantity of lactate efflux from XX/*Sry* Sertoli cells was expected to be lower. To investigate this, Sertoli cells from XY and XX/*Sry* testes at P21 were cultured and the quantities of lactate in the culture media determined. Lactate in the media of both types of Sertoli cells was found to increase over time (Fig. 3c). As expected, amounts were significantly higher in XY than in XX/*Sry* Sertoli cells, possibly indicating that the activity of lactate supply to germ cells is strongly impacted in XX/*Sry* Sertoli cells.

### Cholesterol production decreased in XX/Sry Sertoli cells

**Figure 4:**
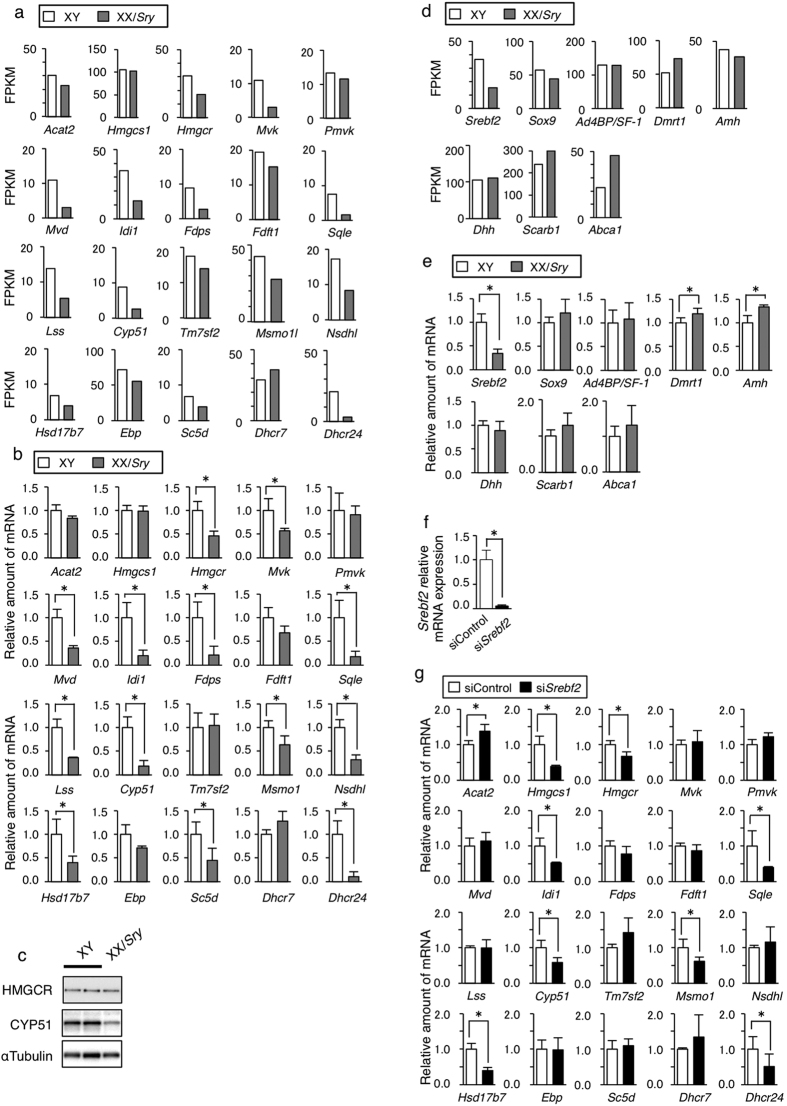
Decreased expression of cholesterogenic genes in XX/*Sry* Sertoli cells. (**a**) FPKM values of cholesterogenic genes obtained from mRNA sequencing. (**b**) Cholesterogenic gene expression was examined by qRT-PCR with RNAs prepared from XY and XX/*Sry* Sertoli cells. The average values for the XY Sertoli cells were normalized to 1.0. (**c**) The amounts of HMGCR and CYP51 in XY and XX/*Sry* Sertoli cells were examined with immunoblotting. α-Tubulin was used as the control. (**d**) FPKM values of *Srebf2, Sox9, Ad4BP/Sf1, Dmrt1, Amh,* and *Dhh, Scarb1,* and *Abca1* are shown. (**e**) *Srebf2, Sox9, Ad4BP/Sf1, Dmrt1, Amh, Dhh, Scarb1,* and *Abca1* were examined by qRT-PCR. The average values for the XY Sertoli cells were normalized to 1.0. (**f**) XY Sertoli cells were treated with siRNA against *Srebf2* and control siRNA. The amount of *Srebf2* was determined by qRT-PCR. (**g**) Expression of cholesterogenic genes in XY Sertoli cells treated with siRNA against *Srebf2* and control siRNA was examined by qRT-PCR. Average values and SDs are indicated. The average values for the siControl-treated cells were normalized to 1.0. Three biologically independent samples (n = 3) were used for the qRT-PCR studies in (**b**,**e**,**f** and **g)**. *p < 0.05.

Sertoli cells supply cholesterol to germ cells. The metabolism, influx, and efflux of cholesterol in or from Sertoli cells are therefore critical for germ cell development. Biological functions related to cholesterol and sterol metabolism were found to be associated with genes that were down-regulated in P21 XX/*Sry* Sertoli cells. In fact, sequence data indicated that the expression of many cholesterogenic genes was down-regulated (Fig. 4a). Similarly, qRT-PCR analysis showed that the expression of 13 out of 20 cholesterogenic genes was significantly decreased in XX/*Sry* Sertoli cells (Fig. 4b). Consistent with the results above, immunoblot studies revealed the amount of CYP51 protein decreased in XX/*Sry* Sertoli cells. Unexpectedly, however, the amount of HMGCR was unchanged (Fig. 4c). Since the amount of HMGCR is regulated post-translationally^36^, HMGCR might be stabilized in XX/*Sry* Sertoli cells.

SREBP2 (sterol regulatory element binding protein 2), also known as SREBF2, has been established as the master regulator of cholesterogenic gene transcription^37^,^38^. Our results obtained by sequencing (Fig. 4d) and qRT-PCR analysis (Fig. 4e) showed a significantly decreased expression of *Srebf2* but unaffected expressions of *Sox9, Ad4BP/SF-1, Dmrt1, Amh,* and *Dhh* in XX/*Sry* Sertoli cells. We consequently investigated whether the decreased expression of *Srebf2* led to a decrease in cholesterogenic gene expression in XY Sertoli cells. siRNA treatment successfully decreased the expression of *Srebf2* (Fig. 4f). qRT-PCR of cholesterogenic genes revealed that eight genes were suppressed by the treatment (Fig. 4g). Seven of these (*Hmgcr, Idi1, Sqle, Cyp51, Msmo1, Hsd17b7*, and *Dhcr24*) were among the genes down-regulated in XX/*Sry* Sertoli cells, suggesting that the down-regulation of the cholesterogenic genes was primarily the result of the decreased expression of *Srebf2*.

Influx and efflux as well as synthesis should be considered in cholesterol homeostasis. Influx of cholesterol into Sertoli cells is predominantly mediated by HDL receptor/SRB1 encoded by *Scarb1*, while efflux is mediated by ABCA1 encoded by *Abca1*^16^,^39^. Sequencing and qRT-PCR analysis indicated that the expression of *Scarb1* was not affected and that *Abca1* expression was unlikely to be down-regulated in XX/*Sry* Sertoli cells (Fig. 4d,e).

**Figure 5:**
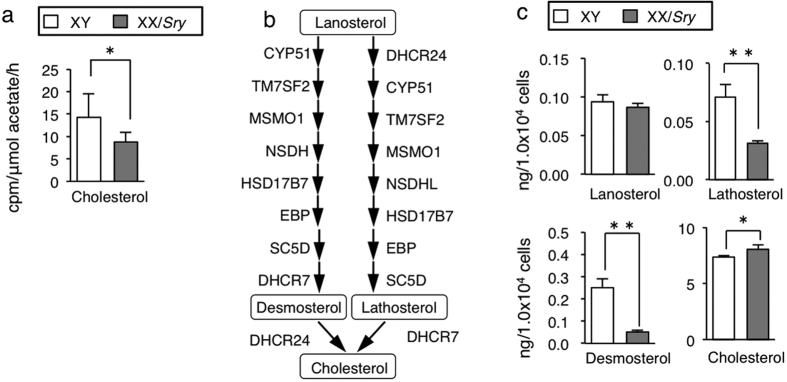
Cholesterol synthesis affected in XX/*Sry* Sertoli cells. (**a**) XY and XX/*Sry* Sertoli cells (1 × 10^6^) were cultured in the presence of [1,2-^14^C]-acetate and the quantities of labeled free and esterified cholesterol were determined. The studies were performed with nine biologically independent XY Sertoli cells (n = 9), and five XX/*Sry* Sertoli cells (n = 5). Error bars indicate SDs. *p < 0.05 (**b**) The late pathway for cholesterol synthesis is shown. (**c**) Quantities of lanosterol, lathosterol, desmosterol, and cholesterol in XY and XX/*Sry* Sertoli cells (3 × 10^4^) were determined. Average values and SDs are indicated. Four biologically independent Sertoli cell samples (n = 4) were used. *p < 0.05; **p < 0.01.

Since these results strongly suggested that cholesterol synthesis is affected in XX/*Sry* Sertoli cells, we investigated cholesterogenic activity. The amount of ^14^C-labeled free cholesterol in cultured XX/*Sry* Sertoli cells was 60% of that in XY Sertoli cells (Fig. 5a). We also determined the quantities of cholesterol together with lanosterol, lathosterol, and desmosterol, all of which are intermediate molecules in the cholesterogenic pathway (Fig. 5b). As expected, the amounts of lathosterol and desmosterol were substantially smaller in XX/*Sry* Sertoli cells (Fig. 5c). Unexpectedly, this was not the case for cholesterol. This may be because the germ cells had mostly disappeared from the XX/*Sry* testes by P21 and the Sertoli cells had thus lost the cells to which they would have transferred their cholesterol.

### Differential epigenetic regulation in XY and XX/Sry Sertoli cells

**Figure 6:**
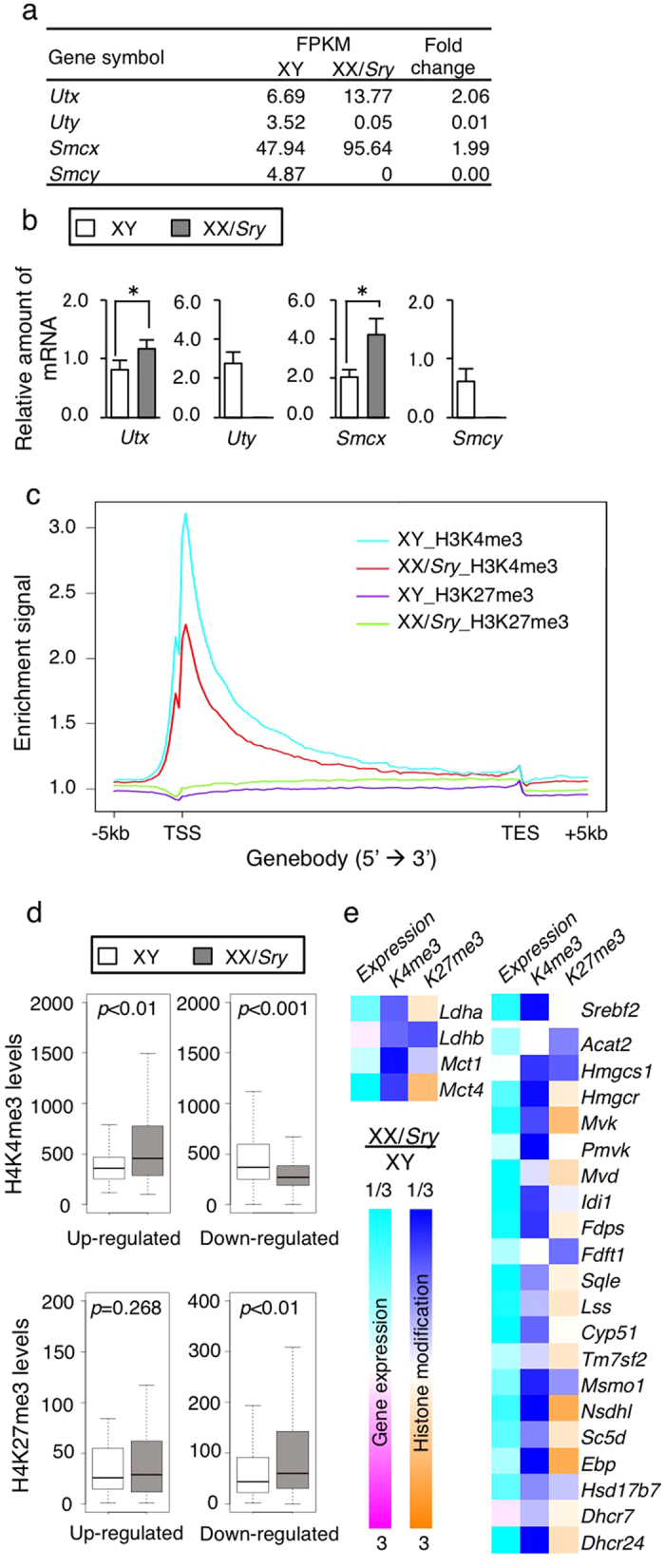
Differential status of histone methylation in XY and XX/*Sry* Sertoli cells. (**a**) Expression levels (FPKM) of *Utx, Uty, Smcx* and *Smcy*, revealed by mRNA sequencing for XY and XX/*Sry* Sertoli cells at P21. (**b**) *Utx, Uty, Smcx* and *Smcy* expression in XY and XX/*Sry* Sertoli cells at P21 was examined by qRT-PCR. The amount of mRNA relative to Rn18s (encoding 18 S ribosomal RNA) is indicated. Three biologically independent samples (n = 3) were used for the qRT-PCR studies. *p < 0.05. (**c**) Global H3K4me3 and H3K27me3 deposition in XY and XX/*Sry* Sertoli cells at P21. Profiles of H3K4me3 and H3K27me3 are represented as normalized read-density. (**d**) Levels of H3K4me3 and H3K27me3 for 3-fold up- or down-regulated genes in XX/*Sry* Sertoli cells. *P* values were computed using the Welch *t*-test. (**e**) Fold changes in gene expression (left), H3K4me3 (middle) and H3K27me3 (right) deposition around the genes involved in lactate and cholesterol metabolism. Magenta and cyan indicate up- and down-regulated genes in XX/*Sry* Sertoli cells, respectively. Orange and blue indicate increased and decreased deposition of histone modification (H3K4me3 and H3K27me3) in XX/*Sry* Sertoli cells relative to XY Sertoli cells. Color gradients correspond to fold change; darker colors indicate a greater degree of change.

Since *Uty* and *Smcy* are localized on the Y chromosome, transcripts of these genes were undetectable in XX/*Sry* Sertoli cells by either sequencing or qRT-PCR (Fig. 6a,b). The expression of *Utx* and *Smcx* was roughly consistent with the gene dosage (a single copy in XY and two copies in XX/*Sry* Sertoli cells). This dosage-dependent expression is consistent with the observation that these genes escape from X chromosome inactivation^40^,^41^. Interestingly, the FPKM value of *Smcx* was 10-fold higher than that of *Smcy*, suggesting that the demethylation activity of H3K4me3 was stronger in XX/*Sry* than in XY Sertoli cells, assuming that its protein products could mediate demethylation with similar enzyme specific activity.

These differential expressions of histone modification enzymes raise the possibility that the methylation status of H3K4 and H3K27 was different in the two types of Sertoli cells. To examine this, we performed genome-wide ChIP-sequencing of both types of Sertoli cells at P21 using the antibodies for H3K4me3 and H3K27me3. H3K4me3 was found to be accumulated around the TSS of genes (Fig. 6c). As expected, the accumulation was substantially lower in XX/*Sry* Sertoli cells, possibly due to higher expression of *Smcx*. The accumulation of H3K27me3 distributed along the gene body was slightly higher in XX/*Sry* than XY Sertoli cells.

We then examined whether the differential status of active H3K4me3 and suppressive H3K27me3 was relevant to the differential gene expression in XY and XX/*Sry* Sertoli cells. As expected, the accumulation of H3K4me3 was greater at the TSS of genes that were up-regulated in XX/*Sry* Sertoli cells (*p* < 0.01), while accumulation was lower around down-regulated genes (*p* < 0.001; Fig. 6d). Accumulation of H3K27me3 was greater around genes that were down-regulated in XX/*Sry* Sertoli cells (*p* < 0.01), while there was no significant accumulation around up-regulated genes (*p* = 0.268).

The expression of *Ldha* and *Mct4*, implicated in the supply of lactate to germ cells, was down-regulated in XX/*Sry* Sertoli cells as described above (Fig. 3b). Consistent with this finding, the accumulation of active H3K4me3 and suppressive H3K27me3 was smaller and greater, respectively, at both *Ldha* and *Mct4* gene loci (Fig. 6e). As noted above, the expression of 13 genes involved in cholesterogenesis was down-regulated in XX/*Sry* Sertoli cells (Fig. 4b). H3K4me3 was decreased to varying degrees around most cholesterogenic genes except *Acta2, Mvd*, and *Fdft1*. An increased tendency for H3K27me3 accumulation was observed in more than half of the cholesterogenic genes.

These histone modifications (decreased H3K4me3 and increased H3K27me3) probably lead to the down-regulation of gene expression. In fact, among the 13 cholesterogenic genes down-regulated in XX/*Sry* Sertoli cells, such changes were observable in *Hmgcr, Mvk, Fdps, Sqle, Lss, Nsdhl, Sc5d*, and *Dhcr24*. As described above, we examined which genes were affected by the down-regulation of *Srebf2* in XX/*Sry* Sertoli cells, and to our surprise found that the expression of *Mvk, Mvd, Fdps, Lss, Nsdhl*, and *Scd5d* was not subject to down-regulation as a result of *Srebf2* knockdown. The chromatin state of all these gene loci (with the exception of *Mvd*) was changed from active to inactive as a result of the decrease in H3K4me3 and increase in H3K27me3. These epigenetic changes might induce the down-regulation of gene expression independently of SREBF2 function in XX/*Sry* Sertoli cells. It should also be noted that the accumulation of H3K4me3 was lower in *Srebf2*, possibly causing the down-regulation of cholesterogenic genes.

## Discussion

To achieve a better understanding of the functional differences between XY and XX/*Sry* Sertoli cells, we compared the mRNA expression profiles of the two cell types and determined which gene expressions were up- or down-regulated in XX/*Sry* Sertoli cells. GO and KEGG pathway analyses suggested that lactate and cholesterol metabolisms are impaired in XX/*Sry* Sertoli cells. Considering that the function of Sertoli cells is the nursing of developing germ cells, these effects on metabolic pathways are intriguing.

The energy metabolic pathway functioning in male germ cells is known to change during the course of differentiation from spermatogonia to spermatozoa^11^. Spermatogonia use glucose as fuel for ATP production, spermatocytes begin to utilize lactate, and later-stage germ cells such as spermatids and spermatozoa are highly depend on lactate as an energy source^12^,^13^. The carbohydrate metabolism of Sertoli cells is unusual; only 25% of the pyruvate produced by glycolysis is oxidized by the TCA cycle^42^, and cultured Sertoli cells mediate reactions from glucose to lactate via pyruvate^43^. This lactate is thought to be supplied to the developing germ cells.

The present study demonstrates that the expression of genes related to lactate metabolism differs between XY and XX/*Sry* Sertoli cells. Quantitative studies have found that *Ldha* is down-regulated in XX/*Sry* Sertoli cells, suggesting the possibility that lactate supply to developing germ cells is reduced in XX/*Sry* testes. This might be supported by the transplantation study of XY germ cells into XX/*Sry* testes performed by Ishii *et al*.^6^. Although the transplanted XY germ cells were capable of completing meiosis, their differentiation into elongated spermatids was impaired in the milieu established by the XX/*Sry* Sertoli cells. Considering that the predominant energy fuel shifts from glucose to lactate at the spermatocyte or spermatozoon stage, it can be assumed that a lower supply of lactate impedes the differentiation of the spermatocyte into an elongated spermatid.

Another crucial nursing function of Sertoli cells is thought to be the supply of cholesterol to germ cells^14^. Cholesterol homeostasis in Sertoli cells is preserved in several ways, such as *de novo* synthesis, influx via the HDL receptor, and efflux via the ABCA1 transporter. Perturbation of cholesterol homeostasis by disruption of *Abca1* resulted in significantly affected spermatogenesis and fertility, in addition to abnormal lipid accumulation in the Sertoli cells^18^.

Our study demonstrated that expression of 13 of the 20 cholesterogenic genes was significantly down-regulated in XX/*Sry* Sertoli cells. Probably because of this suppressed gene expression, *de novo* cholesterol synthetic activity was lower in XX/*Sry* than in XY Sertoli cells. Expression of *Abca1* and *Scarb1* (involved in influx and efflux of cholesterol) was not markedly affected, suggesting that cholesterol transfer remains normal in XX/*Sry* Sertoli cells and that they consequently should feature lower quantities of intracellular cholesterol. However, no difference in the amount of free and esterified cholesterol in the two Sertoli cell types was found. While we are unable to suggest a plausible explanation for this apparent contradiction, the disappearance of germ cells from XX/*Sry* testes might affect the functioning of the XX/*Sry* Sertoli cells by removing the targets of their cholesterol transfer, thus preventing a decrease in their cholesterol content.

Determining the causes of the differential expression of metabolic genes in XX/*Sry* Sertoli cells is an important aim of this research. The cholesterogenic gene *Srebf2,* encoding an already-known key factor for cholesterogenic gene regulation^37^,^38^, was found to be down-regulated in XX/*Sry* Sertoli cells, suggesting a knockdown experiment to determine whether down-regulated *Srebf2* results in reduced expression of cholesterogenic genes. This experiment confirmed the decreased expression of seven of the 13 genes whose expression was down-regulated in XX/*Sry* Sertoli cells. It was therefore assumed that the remaining six genes were regulated by a mechanism independent of SREBF2.

A candidate for the regulation mechanism was suggested by the fact that the genes encoding histone modification enzymes are localized on the sex chromosomes. SMCX on the X and SMCY on the Y chromosome mediate the demethylation of an active histone mark, H3K4me3^21^. UTX mediates the demethylation of a suppressive histone mark, H3K27me3, whereas UTY does not exhibit this activity^22^,^23^. Because of the differential expression of these genes in XY and XX/*Sry* Sertoli cells, it was assumed that the methylation statuses of H3K4 and H3K27 were affected accordingly. Importantly, these epigenetic changes occurred in many, if not all, of the cholesterogenic genes, and we therefore suspect that they cause the down-regulation of cholesterogenic gene expression in XX/*Sry* Sertoli cells.

These observations may support the idea that *Smcx*/*Smcy* and *Utx*/*Uty*, which are localized on the sex chromosomes, regulate sexually dimorphic gene expression. Indeed, a *Utx* gene knockout study demonstrated that UTX regulates the level of H3K27me3, suggesting that the difference in *Utx* gene dosage between the two sexes leads to sex-dependent deposition of H3K27me3^29^. Sex-dependent differences in deposition of H3K27me3 were also identified in hepatocytes and primordial germ cells^44^,^45^.

Gene expression in Sertoli cells is regulated by the testosterone and androgen receptor, AR (NR3C4)^46^. Upon ligand binding, AR forms protein complexes with histone modification enzymes, thereby changing chromatin structure to regulate target gene expression^47^,^48^. Plasma testosterone in XX/*Sry* mice was found to be lower than that in XY males. Although testosterone did not completely disappear from XX/*Sry* mice, this decrease may lead to a suppression of AR target gene expression. Because it is unclear whether the genes involved in lactate and cholesterol metabolism are targets of AR, we cannot exclude the possibility that the decreased expression of those genes is due to the decreased testosterone. Identification of AR target genes in Sertoli cells would be needed to resolve this issue.

In summary, we examined the functional differences between XY and XX/*Sry* Sertoli cells and demonstrated that lactate and cholesterol metabolism, both of which play a crucial role in the nursing of developing germ cells, are down-regulated in XX/*Sry* Sertoli cells. Moreover, our results suggest that these differential functions are at least in part the result of differential expression of histone modification enzymes encoded by sex chromosomes. Although it is well known that XX testes cannot support the differentiation of germ cells even if they carry XY chromosomes, the reason for this has remained unclear. This study suggests that this phenomenon may be caused by the down-regulation of lactate and cholesterol metabolism resulting from altered epigenetic modification.

## Methods

### Mice

A line of XX sex-reversed mice was established using an *Sry* transgene driven by the basal promoter of the *Hsp70.3* gene^49^. The presence of the transgene in mice was confirmed by PCR with primers for *Sry* (Supplemental Table S8). Genetic sex (XY or XX) was determined by PCR using the primers for *Ube1*. Sox9-EGFP knock-in mice have been reported previously^50^. To label XX/*Sry* Sertoli cells with EGFP, XX/*Sry* mice were mated with Sox9-EGFP mice. All protocols for the animal experiments were approved by the Animal Care and Use Committee of Kyushu University. All experiments were performed in accordance with the guidelines.

### Immunofluorescence microscopy

Frozen sections prepared from testes were used for immunostaining^51^. Anti-EGFP rat monoclonal antibody (1:1000; Nacalai Tesque, Kyoto, Japan) and anti-SOX9 rabbit antiserum^52^ (1:2000) were used as the primary antibodies, and ALEXA Fluor 488 goat anti-rat IgG and ALEXA Fluor 555 goat anti-rabbit IgG (1:500; Thermo Fisher Scientific, Waltham, MA, USA) were used as the secondary antibodies. DAPI (4′6′-diamidino-2-phenylindole) was used for nuclear staining. The specimens were observed using a BZ-9000 microscope (Keyence, Osaka, Japan).

### Preparation and culture of Sertoli cells

Testes at postnatal days 1 and 21 (P1 and P21) were incubated in Earle’s balanced salt solution (Sigma, St. Louis, MO, USA) containing 1 mg/ml collagenase (Thermo Fisher Scientific), 0.5 mg/ml dispase (Thermo Fisher Scientific) and 2.5 mg/ml trypsin (Sigma) at 34 °C for 30 min, and following the addition of 0.3 mg/ml deoxyribonuclease I (Roche, Basel, Switzerland), were incubated for another 30 min at 34 °C. After centrifugation, the cells were suspended in PBS with 7-amino-actinomycin D (7AAD; BD Bioscience, Franklin Lakes, NJ, USA). The cells were washed with PBS containing 0.3 mg/ml deoxyribonuclease I, and filtered using a 70 μm cell strainer (BD Bioscience). EGFP-positive Sertoli cells were isolated by fluorescence-activated cell sorting (FACS) using a JSAN cell sorter (Bay bioscience, Kobe, Japan). For siRNA treatment, Sertoli cells at P21 were cultured at 34 °C on 24-well culture plates (Asahi Glass, Tokyo, Japan) pre-coated with collagen type I (Cell Matrix I-C; Nitta Gelatin, Osaka, Japan) in DMEM/Ham’s F-12 (1:1; Nacalai Tesque) supplemented with 10% FBS and Penicillin-streptomycin-glutamine (Thermo Fisher Scientific).

### siRNA treatment

Using an RNeasy Mini or Micro kit (Qiagen, Hilden, Germany), total RNA was prepared from XY and XX/*Sry* Sertoli cells, and XY Sertoli cells were treated with siRNA duplex (stealth RNAi™; Srebf2-MSS277288 or Negative Control Medium GC Duplex; Thermo Fisher Scientific) using Lipofectamine RNAiMAX reagent (Thermo Fisher Scientific) for 48 h.

### Quantitative RT-PCR (qRT-PCR)

Total RNAs were prepared from Sertoli cells at P1 and P21, and from testes and ovaries at P21. They were subjected to cDNA synthesis using M-MLV reverse transcriptase (Thermo Fisher Scientific), and then to qRT-PCR using SYBR Select Master Mix (Thermo Fisher Scientific) and the CFX96 Touch Real-Time PCR Detection System (Bio-Rad Laboratories, Hercules, CA, USA). The primers used for qRT-PCR are listed in Supplemental Table S8. Listed values were standardized using β-actin (*Actb*) or 18 s ribosomal RNA (*Rn18s*). RT-PCR was performed in biological triplicate. Data are presented as means ± 1 standard deviation (SD). Differences between experimental groups were tested for significance using a two-tailed Student’s *t*-test.

### mRNA sequencing and data analyses

Poly(A)^+^ RNAs were prepared from XY and XX/*Sry* Sertoli cells using oligo (dT) magnetic beads. Preparation of mRNA-seq library and subsequent sequencing was carried out as described previously^53^. Mapping and quantification of gene expression was performed by Tophat^54^, version 2.0.8, and RSEM^55^, version 1.2.11, respectively. Expression levels of genes were represented using the number of fragments per kilobase of transcript per million fragments mapped^56^ (FPKM). Fold change in FPKM values in XX/*Sry* Sertoli cells relative to XY Sertoli cells was calculated. Gene sets were subjected to gene ontology^57^ (GO) and KEGG pathway analyses using DAVID^58^. mRNA-seq data have been deposited in DDBJ/EMBL/GeneBank under accession code DRA004090.

### Immunoblot analysis

Whole cell lysates were prepared from XY and XX/*Sry* Sertoli cells using lysis buffer (50 mM Tris-HCl pH 8.0, 50 mM NaCl, 1 mM EDTA, and 1% Sodium Dodecyl Sulfate (SDS)). After the protein concentration was determined using a BCA™ Protein Assay Kit (Pierce Biotechnology, Rockford, IL), 10 μg of the whole cell lysates were subjected to SDS-polyacrylamide gel electrophoresis, followed by immunoblotting. Anti-LDHA (Cell Signaling Technology, Danvers, MA, 1:1000), anti-HMGCR (Abcam, Cambridge, MA, 1:1000), anti-CYP51 (1:1000)^59^, or anti-α tubulin antibody (T-9026, Sigma-Aldrich, St. Louis, MO, 1:1000) was used as the primary antibodies. Anti-rabbit donkey IgG (1:1000) and anti-mouse donkey IgG (GE Healthcare, Piscataway, NJ, 1:1000) were used as the secondary antibodies. Bound antibodies were detected using the Chemi-Lumi One L Western Blotting Detection System (Nacalai Tesque, Kyoto, Japan).

### Chromatin immunoprecipitation-sequence (ChIP-seq)

A total of 10^6^ Sertoli cells fixed by formaldehyde (0.5%, 5 min at room temperature) were lysed with 600 μl Lysis Buffer (5 mM HEPES (pH 8.0), 200 mM KCl, 1 mM CaCl_2_, 1.5 mM MgCl_2_, 5% Sucrose, 0.5% Triton X-100), and then sonicated (5 × 15-s pulses with 59-s break intervals) using the Bioruptor plus sonication device (Diagenode, Denville, NJ, USA). Samples were then digested with 100 U/ml micrococcal nuclease (MNase; Takara Bio, Shiga, Japan) at 37 °C for 1 h to shear the chromatin. MNase digestion was terminated by the addition of 5 mM EDTA (pH 8.0). The sheared chromatin fraction was centrifuged at 15,000 × *g* for 10 min to remove insoluble materials. Supernatant was then incubated overnight at 4 °C with magnetic beads (Dynabeads Protein A; Veritas, Tokyo, Japan) pre-bound with a mouse monoclonal antibody against H3K4me3 or H3K27me3. The beads were washed with Lysis Buffer, Wash Buffer 2 (5 mM HEPES (pH 8.0), 500 mM KCl, 1 mM CaCl_2_, 5% Sucrose, 0.5% NP-40), and Wash Buffer 3 (10 mM Tris-HCl (pH 8.0), 1 mM EDTA (pH 8.0)). Finally, chromatin fractions (ChIP fractions) were eluted from the beads with 50 mM Tris-HCl (pH 8.0), 10 mM EDTA (pH 8.0), and 1% SDS. After crosslinking was reverted by heating at 65 °C for 16 h, DNA fragments were purified using QIAquick PCR Purification kit (Qiagen), and used to prepare a ChIP-seq library with TruSeq ChIP Sample Preparation Kit (Illumina, San Diego, CA, USA). Adaptor-ligated DNA fragments 250 bp in length were recovered. The ChIP-seq library was subjected to sequencing with a HiSeq 2000 (Illumina). Total DNA fragments prepared from the shared chromatin fraction (input fraction) were sequenced as the control. ChIP-seq data have been deposited in DDBJ/EMBL/GeneBank under the accession code DRA004110.

### Analysis of ChIP-seq data sets

ChIP-seq reads were aligned to the reference mouse genome (mm10) using Bowtie^60^, version 1.0.0. The multiple-hit reads were excluded, and only the uniquely mapped reads to the reference mouse genome were kept for further analysis. The number of ChIP-seq reads for H3K4me3 mapped around the transcription start site (TSS; 2 kb upstream to 2 kb downstream) was counted using BEDTools^61^, version 2.17.0. For the analysis of H3K27me3, the number of reads mapped from 2 kb upstream of the TSS to the transcription termination site (TTS) was counted. The number of reads was normalized by the total number of mapped reads to obtain RPM (reads per million). Enrichment values for H3K4me3 and H3K27me3 were defined for each gene as the ratio of RPM in the ChIP fraction to that in the input fraction. Read density profiles of H3K4me3 and H3K27me3 were generated as described previously^62^.

### Measurement of metabolites

Sertoli cells (5 × 10^5^) at P21 were cultured for 3 days. The lactate concentration of 30 μl of the medium was determined at 6 and 24 h after medium change using the Lactate Assay Kit (BioVision, Milpitas, CA, USA). To determine cholesterol synthetic activity, Sertoli cells (1 × 10^6^) were incubated in a serum-free medium containing [1,2–^14^C]-acetate (PerkinElmer, Inc., Boston, MA USA), 50 μM aminoglutethimide (Sigma) and 2 μg/ml 58–035 (ACAT2; acyl-CoA cholesterol acyltransferase inhibitor; Sigma) for 2.5 or 3 h at 34 °C. All lipids, including cholesterol, were extracted using chloroform/methanol (2:1, v/v), then separated by thin-layer chromatography on silica gel with benzene-ethylacetate (2:3, v/v) as a solvent. The radioactivity of a spot containing free and esterified cholesterol visualized with iodine vapor was determined by liquid scintillation counting. The extent of [1,2-^14^C]-acetate incorporation into the cholesterol was expressed as cpm/μmol acetate/h.

### Measurement of quantities of cholesterol and cholesterol precursors

Sertoli cells (3 × 10^4^) were suspended in 0.2 ml methanol and sonicated (2 × 20-s pulses with 30-s break intervals) using Bioruptor Plus (Diagenode), then centrifuged for 5 min at 15,000 rpm to exclude methanol-insoluble cellular components. The supernatant (methanol-soluble fraction) was recovered and evaporated to remove the methanol. Gas chromatography-mass spectrometry analysis (GC-MS) was performed using an Agilent 6890 Plus gas chromatograph interfaced with a single-quadrupole Agilent 5975 C MSD (Agilent Technologies, Palo Alto, CA, USA) as previously described^63^.

### Determination of testosterone, FSH, and LH concentrations

Blood plasma samples collected individually from 6 XY and 6 XX/*Sry* mice at P21 were subjected to LC-MS/MS analysis to determine testosterone concentration. The measurement was performed according to a previous report^64^. Briefly, plasma samples were spiked with ^13^C_3_-testosterone and extracted with 1 mL of 90% hexane/10% ethyl acetate (v/v). After evaporation, the samples were reconstituted in 90% methanol/10% H2O (v/v) for LC-MS/ MS analysis. The samples were analyzed on a QTRAP 5500 LC-MS/MS system (AB SCIEX, Framingham, MA) connected to a Shimadzu LC 20 A HPLC system. For determination of FSH and LH concentration, blood plasma samples were collected individually from 7 XY and 6 XX/*Sry* mice at P21. Plasma FSH and LH concentrations were determined using Rodent FSH ELISA Test Kits and Rodent LH ELISA Test Kits (Endocrine technologies, Inc., Newark, CA), respectively, according to the manufacturer’s instructions.

### Statistical analysis

All experiments were performed with at least three biologically independent samples. Data are presented as the mean and standard deviation. The number of the sample is indicated with ‘n’ in figure legends. The statistical significance was examined using a two-tailed Student’s *t*-test.

## Additional Information

**How to cite this article**: Shishido, Y. *et al*. Differential lactate and cholesterol synthetic activities in XY and XX Sertoli cells. *Sci. Rep.* **7**, 41912; doi: 10.1038/srep41912 (2017).

**Publisher's note:** Springer Nature remains neutral with regard to jurisdictional claims in published maps and institutional affiliations.

## Supplementary Material

Supplemental Tables
