# Supplementary material for: Differential lactate and cholesterol synthetic activities in XY and XX Sertoli cells
Source: Sci Rep. 2017 Feb 2;7:41912. doi: 10.1038/srep41912 (PMC5288785; doi:10.1038/srep41912)
Supplement: Supplemental Tables [file srep41912-s1.pdf]

Differential lactate and cholesterol synthetic activities in XY and XX Sertoli cells

Yurina Shishido, Takashi Baba, Tetsuya Sato, Yuichi Shima, Kanako Miyabayashi, Miki Inoue, Haruhiko Akiyama, Hiroshi Kimura, Yoshiakira Kanai, Yasuhiro Ishihara, Shogo Haraguchi, Akira Miyazaki, Damjana Rozman, Takeshi Yamazaki, Man-Ho Choi, Yasuyuki Ohkawa, Mikita Suyama, Ken-ichirou Morohashi

Supplemental Table S1. Quality data of mRNA-Seq

	Total reads	Mapped reads	Mapping rate	Number of genes detected
**P1_XYSC**	33,411,295	32,539,665	97.4%	16,912
**P1_XX/*Sry*SC**	34,885,622	33,946,217	97.3%	16,955
**P21_XYSC**	29,846,366	29,042,222	97.3%	17,617
**P21_XX/*Sry*SC**	32,676,242	31,787,030	97.3%	16,649

Supplemental Table S2. Genes up-regulated in XX/*Sry* Sertoli cells at P1

Gene symbol	FPKM		Fold change	Gene name
	XY	XX/*Sry*		
Sry	0.0	46.6	1,552.33	sex determining region of Chr Y
Xist	0.0	29.1	726.25	inactive X specific transcripts
Meg3	3.3	8.6	2.66	maternally expressed 3
Ccl27b	15.0	33.7	2.25	chemokine (C-C motif) ligand 27b
Cd24a	4.8	10.6	2.21	CD24a antigen
Clk1	55.9	114.9	2.05	CDC-like kinase 1
Hamp2	9.0	17.4	1.95	hepcidin antimicrobial peptide 2
Kdm6a	10.2	18.9	1.85	lysine (K)-specific demethylase 6A
Arglu1	27.1	48.5	1.79	arginine and glutamate rich 1
Neat1	8.9	15.8	1.77	nuclear paraspeckle assembly transcript 1 (non-protein coding)
Gpc3	9.2	16.3	1.76	glypican 3
Sfrs18	12.7	22.1	1.74	serine/arginine-rich splicing factor 18
Bgn	14.5	25.2	1.74	biglycan
Mgp	17.4	30.1	1.73	matrix Gla protein
Ncam1	7.2	12.3	1.72	neural cell adhesion molecule 1
Paxbp1	11.1	19.0	1.71	PAX3 and PAX7 binding protein 1
6720401G13Rik	15.9	27.1	1.70	RIKEN cDNA 6720401G13 gene
Igfbp7	8.4	14.3	1.70	insulin-like growth factor binding protein 7
Gstt1	10.4	17.7	1.69	glutathione S-transferase, theta 1
Igfbp4	12.7	21.5	1.69	insulin-like growth factor binding protein 4
Tcea2	10.7	18.0	1.68	transcription elongation factor A (SII), 2
Eif2s3x	50.6	84.9	1.68	eukaryotic translation initiation factor 2, subunit 3, structural gene X-linked
Chst11	9.1	15.3	1.68	carbohydrate sulfotransferase 11
Lime1	8.8	14.7	1.67	Lck interacting transmembrane adaptor 1
Fnbp4	18.2	30.0	1.65	formin binding protein 4
Luc7l3	18.3	30.1	1.65	LUC7-like 3 (S. cerevisiae)
Gm1821	11.9	19.5	1.64	ubiquitin pseudogene
Clk4	19.8	32.2	1.63	CDC like kinase 4
Tagln	34.2	55.6	1.63	transgelin
Smoc2	10.0	16.3	1.63	SPARC related modular calcium binding 2
Kcnt1	15.5	25.0	1.62	potassium channel, subfamily T, member 1
Gas5	12.4	19.5	1.57	growth arrest specific 5
Dcn	9.4	14.6	1.56	decorin
Acta2	16.8	26.3	1.56	actin, alpha 2, smooth muscle, aorta
Fstl1	35.8	54.2	1.51	follistatin-like 1
Srsf10	37.0	56.0	1.51	serine/arginine-rich splicing factor 10
Mfap4	14.2	21.3	1.50	microfibrillar-associated protein 4
Srsf11	36.7	55.1	1.50	serine/arginine-rich splicing factor 11

Supplemental Table S3. Genes down-regulated in XX/*Sry* Sertoli cells at P1

Gene symbol	FPKM XY	FPKM XX/*Sry*	Fold change	Gene name
Kdm5d	6.0	0.0	0.00	lysine (K)-specific demethylase 5D
Ddx3y	22.0	0.0	0.00	DEAD (Asp-Glu-Ala-Asp) box polypeptide 3, Y-linked
Eif2s3y	25.6	0.0	0.00	eukaryotic translation initiation factor 2, subunit 3, structural gene Y-linked
Uba1y	10.9	1.0	0.09	ubiquitin-activating enzyme, Chr Y
Dpm3	24.4	3.0	0.12	dolichyl-phosphate mannosyltransferase polypeptide 3
Tppp3	14.1	3.0	0.21	tubulin polymerization-promoting protein family member 3
Gdf15	10.3	2.6	0.25	growth differentiation factor 15
Rpl36	310.4	93.9	0.30	ribosomal protein L36
Rps29	1,080.9	399.7	0.37	ribosomal protein S29
Junb	48.1	18.2	0.38	Jun-B oncogene
1500011K16Rik	8.2	3.2	0.38	RIKEN cDNA 1500011K16 gene
Tceb2	161.9	64.3	0.40	transcription elongation factor B (SIII), polypeptide 2
Smim4	15.6	6.3	0.40	small integral membrane protein 4
Lmna	33.5	14.0	0.42	lamin A
Creb5	13.5	5.8	0.43	cAMP responsive element binding protein 5
Gm8580	11.2	4.9	0.44	ribosomal protein L29 pseudogene
Rnaseh2c	16.3	7.9	0.48	ribonuclease H2, subunit C
Gm5779	11.3	5.5	0.49	ribosomal protein, large, P0 pseudogene
Igsf9b	12.4	6.1	0.49	immunoglobulin superfamily, member 9B
Fosb	232.4	116.5	0.50	FBJ osteosarcoma oncogene B
Esyt3	12.8	6.6	0.51	extended synaptotagmin-like protein 3
Gm9833	15.5	8.2	0.53	myelin basic protein expression factor 2, repressor pseudogene
Nfatc2	12.7	6.7	0.53	nuclear factor of activated T cells, cytoplasmic, calcineurin dependent 2
Hba-a1,Hba-a2	40.3	22.1	0.55	hemoglobin alpha, adult chain 2 | hemoglobin alpha, adult chain 1
Cldn11	40.9	22.5	0.55	claudin 11
Edf1	60.1	33.5	0.56	endothelial differentiation-related factor 1
Hba-a1,Hba-a2	13.7	7.6	0.56	hemoglobin alpha, adult chain 2 | hemoglobin alpha, adult chain 1
Sssca1	21.1	11.8	0.56	Sjogren s syndrome/scleroderma autoantigen 1 homolog (human)
Col9a3	14.0	7.9	0.56	collagen, type IX, alpha 3
Rplp1	740.3	416.3	0.56	ribosomal protein, large, P1
Cidea	39.6	22.6	0.57	cell death-inducing DNA fragmentation factor, alpha subunit-like effector A
Pdlim7	59.2	34.0	0.57	PDZ and LIM domain 7
Nfix	35.5	20.4	0.57	nuclear factor I/X
Jund	40.9	23.5	0.58	Jun proto-oncogene related gene d
Fstl3	58.6	33.7	0.58	follistatin-like 3
Siva1	125.0	72.4	0.58	SIVA1, apoptosis-inducing factor
Tgm2	44.6	25.9	0.58	transglutaminase 2, C polypeptide
Pkig	48.9	28.4	0.58	protein kinase inhibitor, gamma
Pigyl	27.5	16.1	0.58	phosphatidylinositol glycan anchor biosynthesis, class Y-like
Cxx1a	19.2	11.3	0.59	CAAX box 1A
Anxa1	20.5	12.0	0.59	annexin A1
Ier3	260.5	153.9	0.59	immediate early response 3
Arid5a	14.8	8.8	0.59	AT rich interactive domain 5A (MRF1-like)
Csrnp1	60.6	36.2	0.60	cysteine-serine-rich nuclear protein 1
Ncor2	89.3	53.6	0.60	nuclear receptor co-repressor 2
Mrps12	15.3	9.2	0.60	mitochondrial ribosomal protein S12
Cdc42ep5	14.7	8.9	0.61	CDC42 effector protein (Rho GTPase binding) 5
4933409K07Rik	14.9	9.1	0.61	RIKEN cDNA 4933409K07 gene
Col9a2	28.3	17.3	0.61	collagen, type IX, alpha 2
Notch1	16.5	10.2	0.61	notch 1
Sf3b5	40.8	25.1	0.62	splicing factor 3b, subunit 5
Znhit1	14.6	9.1	0.62	zinc finger, HIT domain containing 1

Gene symbol	FPKM XY	FPKM XX/*Sry*	Fold change	Gene name
Beta-s	32.6	20.4	0.63	hemoglobin subunit beta-1-like
Cbln4	22.5	14.1	0.63	cerebellin 4 precursor protein
Ssbp4	35.7	22.5	0.63	single stranded DNA binding protein 4
Sfn	24.4	15.4	0.63	stratifin
Lfng	18.3	11.6	0.63	LFNG O-fucosylpeptide 3-beta-N-acetylglucosaminyltransferase
Dohh	19.1	12.1	0.63	deoxyhypusine hydroxylase/monooxygenase
Lrfn4	20.1	12.8	0.64	leucine rich repeat and fibronectin type III domain containing 4
Padi2	35.6	22.8	0.64	peptidyl arginine deiminase, type II
Fau	681.7	438.8	0.64	Finkel-Biskis-Reilly murine sarcoma virus (FBR-MuSV) ubiquitously expressed (fox derived)
Rps21	800.7	515.8	0.64	ribosomal protein S21
2700094K13Rik	51.8	33.4	0.64	RIKEN cDNA 2700094K13 gene
Bola2	57.2	36.9	0.65	bolA-like 2 (E. coli)
Sh3bp4	30.7	19.8	0.65	SH3-domain binding protein 4
Psmg4	20.5	13.2	0.65	proteasome (prosome, macropain) assembly chaperone 4
Pdlim1	16.0	10.3	0.65	PDZ and LIM domain 1 (elfin)
Cystm1	36.0	23.3	0.65	cysteine-rich transmembrane module containing 1
Fabp3	63.7	41.3	0.65	fatty acid binding protein 3, muscle and heart
Klf3	20.6	13.4	0.65	Kruppel-like factor 3 (basic)
Nr1d1	23.5	15.3	0.65	nuclear receptor subfamily 1, group D, member 1
Ddah2	22.5	14.7	0.65	dimethylarginine dimethylaminohydrolase 2
Sox9	87.4	57.1	0.65	SRY-box containing gene 9
Hexim1	36.2	23.7	0.65	hexamethylene bis-acetamide inducible 1
Arl10	19.4	12.7	0.66	ADP-ribosylation factor-like 10
Dbn1	42.8	28.2	0.66	drebrin 1
Uqcr10	124.5	82.0	0.66	ubiquinol-cytochrome c reductase, complex III subunit X
Arid5b	36.7	24.3	0.66	AT rich interactive domain 5B (MRF1-like)
Myc	17.2	11.4	0.66	myelocytomatosis oncogene
Polr2l	47.4	31.4	0.66	polymerase (RNA) II (DNA directed) polypeptide L
Crym	17.0	11.3	0.66	crystallin, mu
Cxx1b	19.7	13.1	0.66	CAAX box 1B
Galk1	27.2	18.0	0.66	galactokinase 1
S100a10	192.8	128.3	0.66	S100 calcium binding protein A10 (calpactin)
Vps37b	35.7	23.8	0.66	vacuolar protein sorting 37B (yeast)
Ctgf	192.5	128.1	0.66	connective tissue growth factor

Gene symbol	FPKM XY	FPKM XX/*Sry*	Fold change	Gene name
Xist	0.1	62.9	1,048.17	inactive X specific transcripts
4933409K07Rik	0.0	5.1	170.00	RIKEN cDNA 4933409K07 gene
Ins2	0.1	5.7	56.50	insulin II
Lfng	0.1	6.2	56.00	LFNG O-fucosylpeptide 3-beta-N-acetylglucosaminyltransferase
Sel1l3	0.5	18.6	36.55	sel-1 suppressor of lin-12-like 3 (C. elegans)
Tgfbi	1.0	31.4	33.02	transforming growth factor, beta induced
Cadps	0.3	9.0	30.07	Ca2+-dependent secretion activator
Bdh1	0.7	18.1	27.86	3-hydroxybutyrate dehydrogenase, type 1
Pramef12	0.7	15.1	21.61	PRAME family member 12
Plxdc2	0.3	6.3	21.59	plexin domain containing 2
Nrep	1.2	26.2	21.15	neuronal regeneration related protein
Ltbp3	0.6	13.3	21.08	latent transforming growth factor beta binding protein 3
Wls	0.7	14.6	19.72	wntless homolog (Drosophila)
Wfdc18	0.8	14.7	18.41	WAP four-disulfide core domain 18
Pmp22	0.9	15.5	16.53	peripheral myelin protein 22
C1ra	1.3	19.5	15.62	complement component 1, r subcomponent A
Hspb8	1.5	22.7	15.51	heat shock protein 8
Aif1l	0.5	7.1	15.13	allograft inflammatory factor 1-like
Myoz2	2.3	32.8	14.33	myozenin 2
C1s	1.0	13.7	13.18	complement component 1, s subcomponent
Epha2	0.6	6.5	11.75	Eph receptor A2
Gpx8	1.1	12.6	11.69	glutathione peroxidase 8 (putative)
Zmiz1	0.7	7.2	10.83	zinc finger, MIZ-type containing 1
Plekhb1	1.8	18.9	10.65	pleckstrin homology domain containing, family B (evectins) member 1
Ildr2	2.8	27.4	9.77	immunoglobulin-like domain containing receptor 2
Cd9	2.6	24.5	9.53	CD9 antigen
Oxct1	8.3	78.5	9.42	3-oxoacid CoA transferase 1
Tnfrsf12a	10.0	93.6	9.41	tumor necrosis factor receptor superfamily, member 12a
Oaf	1.0	8.9	9.21	OAF homolog (Drosophila)
Dct	1.3	11.4	8.91	dopachrome tautomerase
Adamts1	1.1	9.7	8.85	a disintegrin-like and metallopeptidase (reprolysin type) with thrombospondin type 1 motif, 1
Fabp3	1.8	15.1	8.65	fatty acid binding protein 3, muscle and heart
Slc6a8	4.8	38.9	8.13	solute carrier family 6 (neurotransmitter transporter, creatine), member 8
Tceal6	2.1	15.8	7.51	transcription elongation factor A (SII)-like 6
Gucy1b3	0.8	6.1	7.34	guanylate cyclase 1, soluble, beta 3
Klf3	2.6	18.4	7.22	Kruppel-like factor 3 (basic)
Sec14l2	0.9	6.3	7.17	SEC14-like 2 (S. cerevisiae)
Fam124a	1.1	7.8	7.05	family with sequence similarity 124, member A
Igfbp3	5.5	38.3	6.99	insulin-like growth factor binding protein 3
Tmem37	1.9	13.3	6.97	transmembrane protein 37
Fgfr1	1.8	12.1	6.91	fibroblast growth factor receptor 1
Rassf8	2.3	15.6	6.82	Ras association (RalGDS/AF-6) domain family (N-terminal) member 8
Athl1	1.4	9.2	6.68	ATH1, acid trehalase-like 1 (yeast)
Antxr1	2.2	14.5	6.65	anthrax toxin receptor 1
Cyr61	39.9	264.3	6.62	cysteine rich protein 61
Lbh	1.4	9.0	6.55	limb-bud and heart
Itgb3	2.0	12.6	6.48	integrin beta 3
Tppp3	4.3	27.2	6.35	tubulin polymerization-promoting protein family member 3
Amotl1	3.3	20.0	6.05	angiomotin-like 1
Mgat3	2.5	15.1	6.01	mannoside acetylglucosaminyltransferase 3
Tesc	6.6	39.5	5.98	tescalcin
Fads6	1.4	8.0	5.78	fatty acid desaturase domain family, member 6

Gene symbol	FPKM XY	FPKM XX/*Sry*	Fold change	Gene name
Cxxc5	1.7	9.1	5.51	CXXC finger 5
Npr2	1.4	7.4	5.41	natriuretic peptide receptor 2
Tpm4	2.1	10.9	5.28	tropomyosin 4
Star	7.6	39.8	5.22	steroidogenic acute regulatory protein
Rasl11a	7.3	37.6	5.17	RAS-like, family 11, member A
Filip1l	4.7	24.0	5.09	filamin A interacting protein 1-like
Cybrd1	1.5	7.6	5.07	cytochrome b reductase 1
Pdlim4	13.1	65.9	5.05	PDZ and LIM domain 4
Nbl1	9.3	45.8	4.95	neuroblastoma, suppression of tumorigenicity 1
Serpinh1	3.8	18.4	4.87	serine (or cysteine) peptidase inhibitor, clade H, member 1
Hr	2.6	12.4	4.86	hairless
Pam	4.5	21.8	4.85	peptidylglycine alpha-amidating monooxygenase
Tbc1d2b	3.1	14.7	4.80	TBC1 domain family, member 2B
Scx	3.6	16.6	4.68	scleraxis
Rnf208	1.5	6.9	4.67	ring finger protein 208
D430019H16Rik	1.7	7.9	4.67	RIKEN cDNA D430019H16 gene
Pgm1	2.1	9.6	4.61	phosphoglucomutase 1
Aldh3b1	1.9	8.5	4.56	aldehyde dehydrogenase 3 family, member B1
Masp1	2.2	10.0	4.45	mannan-binding lectin serine peptidase 1
Sema4d	1.5	6.6	4.38	sema domain, immunoglobulin domain (Ig), transmembrane domain (TM) and short cytoplasmic domain, (semaphorin) 4D
Col4a2	11.3	49.1	4.34	collagen, type IV, alpha 2
Cd63	9.1	38.9	4.28	CD63 antigen
E130114P18Rik	1.6	7.0	4.28	RIKEN cDNA E130114P18 gene
Wnt5a	2.3	9.6	4.25	wingless-related MMTV integration site 5A
Syt12	1.7	7.4	4.22	synaptotagmin XII
Crim1	2.7	11.4	4.20	cysteine rich transmembrane BMP regulator 1 (chordin like)
Erbb4	2.8	11.4	4.11	v-erb-a erythroblastic leukemia viral oncogene homolog 4 (avian)
Myadm	3.8	15.8	4.10	myeloid-associated differentiation marker
Tmbim1	2.7	11.1	4.10	transmembrane BAX inhibitor motif containing 1
D1Ertd622e	2.1	8.5	4.07	DNA segment, Chr 1, ERATO Doi 622, expressed
Slc1a6	2.1	8.4	4.05	solute carrier family 1 (high affinity aspartate/glutamate transporter), member 6
Rasgef1c	2.1	8.3	4.05	RasGEF domain family, member 1C
Parva	2.6	10.4	4.04	parvin, alpha
Serpine2	1.7	6.8	3.95	serine (or cysteine) peptidase inhibitor, clade E, member 2
Tceal3	4.2	16.2	3.88	transcription elongation factor A (SII)-like 3
Pcolce	4.9	18.7	3.85	procollagen C-endopeptidase enhancer protein
Epb4.1l1	3.4	12.9	3.80	erythrocyte protein band 4.1-like 1
Tbc1d30	6.9	25.8	3.73	TBC1 domain family, member 30
Cldn10	3.0	10.7	3.62	claudin 10
Kif5c	2.9	10.3	3.60	kinesin family member 5C
Synm	5.9	21.3	3.59	synemin, intermediate filament protein
Itprip	3.7	13.0	3.55	inositol 1,4,5-triphosphate receptor interacting protein
Dcn	2.7	9.6	3.52	decorin
Prkca	2.8	9.7	3.49	protein kinase C, alpha
Smo	8.1	28.0	3.45	smoothened homolog (Drosophila)
Trpc3	4.4	15.0	3.38	transient receptor potential cation channel, subfamily C, member 3
Sec24d	3.7	12.1	3.27	Sec24 related gene family, member D (S. cerevisiae)
Atf3	31.5	102.5	3.25	activating transcription factor 3
Cyp2s1	9.9	32.1	3.25	cytochrome P450, family 2, subfamily s, polypeptide 1
Scara3	24.8	80.5	3.24	scavenger receptor class A, member 3
Itgb8	3.3	10.6	3.23	integrin beta 8
Prrg4	2.6	8.3	3.19	proline rich Gla (G-carboxyglutamic acid) 4 (transmembrane)
Dab2ip	6.9	21.7	3.14	disabled 2 interacting protein
Pyroxd2	2.9	9.2	3.13	pyridine nucleotide-disulphide oxidoreductase domain 2

Gene symbol	FPKM XY	FPKM XX/*Sry*	Fold change	Gene name
Slc27a3	7.8	24.5	3.12	solute carrier family 27 (fatty acid transporter), member 3
Rgs11	8.2	24.8	3.02	regulator of G-protein signaling 11
Dock5	7.1	21.4	3.01	dedicator of cytokinesis 5
Fxyd1	5.1	15.1	2.96	FXYD domain-containing ion transport regulator 1
C1qtnf6	15.5	45.8	2.96	C1q and tumor necrosis factor related protein 6
Clip1	3.6	10.7	2.93	CAP-GLY domain containing linker protein 1
B2m	43.1	125.5	2.92	beta-2 microglobulin
Mras	16.3	46.9	2.89	muscle and microspikes RAS
Slc12a4	14.9	43.0	2.88	solute carrier family 12, member 4
Prkar2b	24.2	69.2	2.86	protein kinase, cAMP dependent regulatory, type II beta
Rhoc	14.2	40.3	2.85	ras homolog gene family, member C
Efemp1	8.5	24.1	2.84	epidermal growth factor-containing fibulin-like extracellular matrix protein 1
Vnn1	4.3	12.1	2.84	vanin 1
Ncmap	11.9	33.5	2.80	noncompact myelin associated protein
Arnt2	4.8	13.5	2.80	aryl hydrocarbon receptor nuclear translocator 2
Slc38a5	36.4	101.8	2.80	solute carrier family 38, member 5
Gsn	3.0	8.3	2.77	gelsolin
Bmf	4.4	12.1	2.77	BCL2 modifying factor
AI848285	3.3	9.1	2.76	expressed sequence AI848285
Grem2	3.8	10.4	2.75	gremlin 2 homolog, cysteine knot superfamily (Xenopus laevis)
Plekha6	3.8	10.2	2.72	pleckstrin homology domain containing, family A member 6
Cd59a	17.0	46.2	2.72	CD59a antigen
Pappa	7.5	20.4	2.71	pregnancy-associated plasma protein A
Ptprd	11.6	31.2	2.70	protein tyrosine phosphatase, receptor type, D
Ptrf	6.3	17.0	2.69	polymerase I and transcript release factor
Grb10	3.3	8.7	2.63	growth factor receptor bound protein 10
Ston1	5.3	13.8	2.62	stonin 1
Padi2	18.4	48.1	2.61	peptidyl arginine deiminase, type II
Bex1	7.3	18.8	2.59	brain expressed gene 1
Tspan4	10.7	27.6	2.58	tetraspanin 4
Gdnf	5.3	13.6	2.57	glial cell line derived neurotrophic factor
Lrch1	6.1	15.7	2.55	leucine-rich repeats and calponin homology (CH) domain containing 1
Syngr1	9.2	23.5	2.55	synaptogyrin 1
Sdpr	8.1	20.7	2.55	serum deprivation response
Ust	4.6	11.7	2.54	uronyl-2-sulfotransferase
Lama2	5.2	13.1	2.54	laminin, alpha 2
Nfil3	20.3	51.1	2.52	nuclear factor, interleukin 3, regulated
Sptb	4.7	11.5	2.48	spectrin beta, erythrocytic
Olig1	4.6	11.3	2.47	oligodendrocyte transcription factor 1
Tgm2	19.9	48.8	2.46	transglutaminase 2, C polypeptide
Hbegf	4.4	10.7	2.44	heparin-binding EGF-like growth factor
Rapgef2	3.6	8.7	2.43	Rap guanine nucleotide exchange factor (GEF) 2
Nedd9	13.5	32.7	2.42	neural precursor cell expressed, developmentally down-regulated gene 9
Ctsz	5.2	12.5	2.42	cathepsin Z
Tpst1	5.4	13.0	2.40	protein-tyrosine sulfotransferase 1
Ets2	4.1	9.9	2.39	E26 avian leukemia oncogene 2, 3 domain
B4galt6	11.0	26.2	2.38	UDP-Gal:betaGlcNAc beta 1,4-galactosyltransferase, polypeptide 6
Gsdmd	4.0	9.6	2.37	gasdermin D
Zfp36l1	5.3	12.5	2.36	zinc finger protein 36, C3H type-like 1
Mb21d2	8.7	20.4	2.34	Mab-21 domain containing 2
Cpd	10.3	24.1	2.34	carboxypeptidase D
Nfatc2	7.3	16.8	2.31	nuclear factor of activated T cells, cytoplasmic, calcineurin dependent 2

Gene symbol	FPKM XY	FPKM XX/*Sry*	Fold change	Gene name
Tmem159	5.4	12.4	2.31	transmembrane protein 159
Tcn2	14.3	32.9	2.31	transcobalamin 2
Bok	11.7	26.9	2.31	BCL2-related ovarian killer protein
Gfpt2	8.8	20.1	2.30	glutamine fructose-6-phosphate transaminase 2
Parp8	4.0	9.1	2.29	poly (ADP-ribose) polymerase family, member 8
Zbtb46	7.5	17.3	2.29	zinc finger and BTB domain containing 46
Anxa6	28.1	64.3	2.29	annexin A6
Cnn3	90.8	204.2	2.25	calponin 3, acidic
Iffo2	10.5	23.5	2.25	intermediate filament family orphan 2
Bnip3	13.9	31.1	2.24	BCL2/adenovirus E1B interacting protein 3
Mef2c	5.4	12.1	2.23	myocyte enhancer factor 2C
Fzd3	5.1	11.4	2.23	frizzled homolog 3 (Drosophila)
Acta2	39.1	86.7	2.22	actin, alpha 2, smooth muscle, aorta
Zfp704	13.3	29.4	2.22	zinc finger protein 704
Fam114a1	5.6	12.3	2.21	family with sequence similarity 114, member A1
Zcchc18	7.6	16.6	2.19	zinc finger, CCHC domain containing 18
Arhgap21	6.8	14.9	2.19	Rho GTPase activating protein 21
Mt3	39.1	85.1	2.18	metallothionein 3
Lphn2	12.5	27.2	2.17	latrophilin 2
Bid	7.1	15.4	2.16	BH3 interacting domain death agonist
Epyc	7.8	16.8	2.15	epiphycan
Eif2s3x	31.2	67.0	2.14	eukaryotic translation initiation factor 2, subunit 3, structural gene X-linked
Cldn7	5.1	10.9	2.14	claudin 7
Klf4	15.7	33.4	2.13	Kruppel-like factor 4 (gut)
Sipa1l1	6.1	13.1	2.13	signal-induced proliferation-associated 1 like 1
Cmtm4	8.9	18.9	2.12	CKLF-like MARVEL transmembrane domain containing 4
Arid5a	9.2	19.4	2.11	AT rich interactive domain 5A (MRF1-like)
Ctif	9.4	19.9	2.11	CBP80/20-dependent translation initiation factor
Prss12	7.2	15.1	2.11	protease, serine, 12 neurotrypsin (motopsin)
Stat5b	8.4	17.8	2.11	signal transducer and activator of transcription 5B
Cmtm7	8.2	17.3	2.10	CKLF-like MARVEL transmembrane domain containing 7
Syt9	13.8	28.7	2.08	synaptotagmin IX
Abca1	22.6	46.9	2.07	ATP-binding cassette, sub-family A (ABC1), member 1
Prss35	52.8	109.0	2.06	protease, serine, 35
Kdm6a	6.7	13.8	2.06	lysine (K)-specific demethylase 6A
Wdfy1	7.2	14.7	2.06	WD repeat and FYVE domain containing 1
Arhgef4	5.0	10.3	2.05	Rho guanine nucleotide exchange factor (GEF) 4
Fkbp10	11.4	23.4	2.05	FK506 binding protein 10
Sept11	29.2	59.8	2.05	septin 11
Nudt19	26.1	53.0	2.03	nudix (nucleoside diphosphate linked moiety X)-type motif 19
Zbtb8os	45.3	91.7	2.03	zinc finger and BTB domain containing 8 opposite strand
Megf9	6.2	12.6	2.02	multiple EGF-like-domains 9
Aida	18.4	37.1	2.02	axin interactor, dorsalization associated
Fam211b	10.2	20.6	2.02	family with sequence similarity 211, member B
Wwtr1	6.8	13.6	2.01	WW domain containing transcription regulator 1
Slk	7.3	14.7	2.00	STE20-like kinase
Kdm5c	47.9	95.6	1.99	lysine (K)-specific demethylase 5C
Fam168a	5.7	11.2	1.99	family with sequence similarity 168, member A
AI414108	7.3	14.5	1.98	expressed sequence AI414108
Retsat	22.1	43.5	1.97	retinol saturase (all trans retinol 13,14 reductase)
Hint1	60.8	119.4	1.97	histidine triad nucleotide binding protein 1
A630077J23Rik	9.7	19.0	1.96	RIKEN cDNA A630077J23 gene
Slc35e3	8.8	17.3	1.96	solute carrier family 35, member E3
Cbfb	17.1	33.4	1.95	core binding factor beta

Gene symbol	FPKM		Fold change	Gene name
	XY	XX/*Sry*		
Pdpn	13.8	27.0	1.95	podoplanin
Fat1	69.2	134.7	1.95	FAT tumor suppressor homolog 1 (Drosophila)
Ubtd2	8.7	16.9	1.94	ubiquitin domain containing 2
Spp1	54.0	104.5	1.94	secreted phosphoprotein 1
Fbxo4	5.5	10.6	1.93	F-box protein 4
Deptor	9.5	18.3	1.93	DEP domain containing MTOR-interacting protein
Oplah	16.3	31.5	1.93	5-oxoprolinase (ATP-hydrolysing)
Lpar1	18.2	35.0	1.92	lysophosphatidic acid receptor 1
Clk1	27.1	52.0	1.92	CDC-like kinase 1
Dnajb4	19.8	37.8	1.91	DnaJ (Hsp40) homolog, subfamily B, member 4
Sptssa	46.7	89.3	1.91	serine palmitoyltransferase, small subunit A
Sspn	6.1	11.6	1.91	sarcospan
Clip3	11.2	21.4	1.91	CAP-GLY domain containing linker protein 3
Syne1	7.4	14.2	1.91	spectrin repeat containing, nuclear envelope 1
Igfbp4	8.5	16.1	1.90	insulin-like growth factor binding protein 4
Robo4	5.8	10.9	1.90	roundabout homolog 4 (Drosophila)
Slc7a2	14.4	27.3	1.90	solute carrier family 7 (cationic amino acid transporter, y+ system), member 2
Iah1	13.2	25.0	1.90	isoamyl acetate-hydrolyzing esterase 1 homolog (S. cerevisiae)
Pm20d1	6.5	12.4	1.90	peptidase M20 domain containing 1
Pdlim3	8.3	15.7	1.89	PDZ and LIM domain 3
Pds5b	6.2	11.7	1.89	PDS5, regulator of cohesion maintenance, homolog B (S. cerevisiae)
Clcn5	14.9	28.2	1.89	chloride channel 5
Lysmd2	10.3	19.4	1.88	LysM, putative peptidoglycan-binding, domain containing 2
Zfp239	8.0	15.0	1.88	zinc finger protein 239
Pak1	10.6	19.8	1.88	p21 protein (Cdc42/Rac)-activated kinase 1
Ntn4	37.7	70.4	1.87	netrin 4
Slc36a4	6.0	11.2	1.87	solute carrier family 36 (proton/amino acid symporter), member 4
Acvr1	7.4	13.9	1.87	activin A receptor, type 1
Nfkb1	9.3	17.3	1.86	nuclear factor of kappa light polypeptide gene enhancer in B cells 1, p105
Ugdh	7.1	13.2	1.86	UDP-glucose dehydrogenase
Trip6	31.9	59.0	1.85	thyroid hormone receptor interactor 6
Ptprm	17.7	32.6	1.85	protein tyrosine phosphatase, receptor type, M
Kif1a	23.6	43.6	1.85	kinesin family member 1A
Ppap2b	12.6	23.3	1.84	phosphatidic acid phosphatase type 2B
Tmem150a	7.6	14.0	1.84	transmembrane protein 150A
Gyg	10.3	18.9	1.83	glycogenin
Ctsb	68.4	124.9	1.83	cathepsin B
Sema5a	13.6	24.7	1.82	sema domain, seven thrombospondin repeats (type 1 and type 1-like), transmembrane domain (TM) and short cytoplasmic domain, (semaphorin) 5A
Mapre2	6.4	11.5	1.80	microtubule-associated protein, RP/EB family, member 2
Tmie	13.1	23.7	1.80	transmembrane inner ear
Tmem62	6.5	11.6	1.80	transmembrane protein 62
Ctsf	62.1	111.4	1.80	cathepsin F
Itga6	44.9	80.6	1.80	integrin alpha 6
Myl12a	93.8	168.3	1.80	myosin, light chain 12A, regulatory, non-sarcomeric
Kitl	45.3	81.3	1.79	kit ligand
Pdgfc	15.3	27.3	1.79	platelet-derived growth factor, C polypeptide
Ppm1l	13.4	23.8	1.78	protein phosphatase 1 (formerly 2C)-like
Cers6	14.2	25.3	1.78	ceramide synthase 6
Fbxo6	8.5	15.1	1.78	F-box protein 6
Ass1	28.0	49.4	1.76	argininosuccinate synthetase 1
Il10rb	29.2	51.4	1.76	interleukin 10 receptor, beta

Gene symbol	FPKM XY	FPKM XX/*Sry*	Fold change	Gene name
Arhgdig	10.4	18.3	1.76	Rho GDP dissociation inhibitor (GDI) gamma
Lrrc8b	21.2	37.1	1.75	leucine rich repeat containing 8 family, member B
1190002N15Rik	8.3	14.6	1.75	RIKEN cDNA 1190002N15 gene
Stat1	36.3	63.3	1.75	signal transducer and activator of transcription 1
Numb	11.9	20.8	1.75	numb gene homolog (Drosophila)
Tmem63a	10.3	18.0	1.74	transmembrane protein 63a
Trib1	8.5	14.8	1.74	tribbles homolog 1 (Drosophila)
Itpk1	29.2	50.7	1.74	inositol 1,3,4-triphosphate 5/6 kinase
Zmat1	17.5	30.3	1.73	zinc finger, matrin type 1
Znrf3	7.1	12.3	1.73	zinc and ring finger 3
Tax1bp3	8.8	15.2	1.72	Tax1 (human T cell leukemia virus type I) binding protein 3
Tpm1	22.4	38.5	1.72	tropomyosin 1, alpha
Edn1	53.0	91.0	1.72	endothelin 1
Chmp7	40.3	69.3	1.72	charged multivesicular body protein 7
Tcf12	17.3	29.7	1.72	transcription factor 12
Dst	13.0	22.3	1.72	dystonin
Klhl2	8.6	14.7	1.71	kelch-like 2, Mayven
Serpinb6a	93.0	159.2	1.71	serine (or cysteine) peptidase inhibitor, clade B, member 6a
Prkar2a	21.3	36.5	1.71	protein kinase, cAMP dependent regulatory, type II alpha
Smg1	14.1	24.0	1.70	SMG1 homolog, phosphatidylinositol 3-kinase-related kinase (C. elegans)
Dbi	200.0	340.5	1.70	diazepam binding inhibitor
Kif1b	8.1	13.7	1.70	kinesin family member 1B
Ppp2r2c	20.1	33.9	1.69	protein phosphatase 2 (formerly 2A), regulatory subunit B (PR 52), gamma isoform
Wdfy3	9.2	15.5	1.69	WD repeat and FYVE domain containing 3
Pde4d	12.6	21.3	1.69	phosphodiesterase 4D, cAMP specific
Ccs	31.4	52.9	1.69	copper chaperone for superoxide dismutase
Ctnnal1	89.2	150.3	1.68	catenin (cadherin associated protein), alpha-like 1
F3	44.0	74.1	1.68	coagulation factor III
Ikbip	8.6	14.5	1.68	IKBKB interacting protein
Plac1	28.6	48.1	1.68	placental specific protein 1
Tomt	8.0	13.5	1.68	transmembrane O-methyltransferase
Kazald1	34.4	57.6	1.68	Kazal-type serine peptidase inhibitor domain 1
Alas1	15.8	26.4	1.67	aminolevulinic acid synthase 1
Brwd1	24.6	41.1	1.67	bromodomain and WD repeat domain containing 1
Xpr1	9.1	15.2	1.67	xenotropic and polytropic retrovirus receptor 1
1700030C10Rik	22.4	37.2	1.66	RIKEN cDNA 1700030C10 gene
Tspan3	94.4	156.6	1.66	tetraspanin 3
Ppm1k	12.5	20.7	1.66	protein phosphatase 1K (PP2C domain containing)
Tmtc2	17.7	29.2	1.66	transmembrane and tetratricopeptide repeat containing 2
Aebp2	36.1	59.7	1.65	AE binding protein 2
Ugp2	14.5	23.9	1.65	UDP-glucose pyrophosphorylase 2
Zfp462	11.4	18.8	1.65	zinc finger protein 462
Prrc2b	17.4	28.7	1.65	proline-rich coiled-coil 2B
Acot1	10.1	16.6	1.64	acyl-CoA thioesterase 1
2610002J02Rik	17.1	28.0	1.64	RIKEN cDNA 2610002J02 gene
Sppl2a	10.7	17.5	1.64	signal peptide peptidase like 2A
Fermt2	34.8	57.0	1.64	fermitin family homolog 2 (Drosophila)
Nfix	15.3	24.9	1.64	nuclear factor I/X
Dusp16	14.0	22.8	1.64	dual specificity phosphatase 16
Slc20a1	19.0	31.0	1.63	solute carrier family 20, member 1
Ano6	30.3	49.4	1.63	anoctamin 6
Arhgap5	12.9	21.1	1.63	Rho GTPase activating protein 5
Klhl15	12.4	20.2	1.63	kelch-like 15
Irf2bp2	17.6	28.7	1.63	interferon regulatory factor 2 binding protein 2

Gene symbol	FPKM XY	FPKM XX/*Sry*	Fold change	Gene name
Man1a2	13.4	21.7	1.62	mannosidase, alpha, class 1A, member 2
Micall1	9.3	15.1	1.62	microtubule associated monoxygenase, calponin and LIM domain containing -like 1
Malat1	40.5	65.7	1.62	metastasis associated lung adenocarcinoma transcript 1 (non-coding RNA)
Snx25	17.2	28.0	1.62	sorting nexin 25
Fkbp3	33.4	54.1	1.62	FK506 binding protein 3
Sh3bgr	9.2	14.9	1.62	SH3-binding domain glutamic acid-rich protein
Clic4	33.0	53.3	1.61	chloride intracellular channel 4 (mitochondrial)
Tspan12	9.1	14.7	1.61	tetraspanin 12
Srr	11.8	18.9	1.61	serine racemase
Nfia	9.4	15.1	1.61	nuclear factor I/A
Phip	10.0	16.1	1.61	pleckstrin homology domain interacting protein
Cmtm3	38.4	61.6	1.60	CKLF-like MARVEL transmembrane domain containing 3
Anxa7	12.9	20.7	1.60	annexin A7
Ifnar2	17.8	28.5	1.60	interferon (alpha and beta) receptor 2
Tspyl2	139.9	223.7	1.60	TSPY-like 2
Zhx2	8.8	14.1	1.60	zinc fingers and homeoboxes 2
Myl12b	136.6	218.3	1.60	myosin, light chain 12B, regulatory
Dip2a	8.8	14.0	1.59	DIP2 disco-interacting protein 2 homolog A (Drosophila)
Morf4l2	180.1	286.8	1.59	mortality factor 4 like 2
Zeb2	8.8	14.0	1.59	zinc finger E-box binding homeobox 2
Acat3	14.8	23.5	1.59	acetyl-Coenzyme A acetyltransferase 3
Usmg5	81.8	130.0	1.59	upregulated during skeletal muscle growth 5
Nav2	11.3	17.9	1.59	neuron navigator 2
Rrm1	9.5	15.0	1.59	ribonucleotide reductase M1
Ctps	15.9	25.1	1.58	cytidine 5 -triphosphate synthase
Ddx3x	84.5	133.6	1.58	DEAD/H (Asp-Glu-Ala-Asp/His) box polypeptide 3, X-linked
Mgea5	43.1	68.1	1.58	meningioma expressed antigen 5 (hyaluronidase)
Dyrk2	41.7	65.8	1.58	dual-specificity tyrosine-(Y)-phosphorylation regulated kinase 2
Ube2e1	27.3	43.1	1.58	ubiquitin-conjugating enzyme E2E 1
Acap2	10.5	16.6	1.58	ArfGAP with coiled-coil, ankyrin repeat and PH domains 2
Gramd1b	11.2	17.7	1.58	GRAM domain containing 1B
Dusp11	21.6	34.0	1.58	dual specificity phosphatase 11 (RNA/RNP complex 1-interacting)
Cnih4	9.6	15.1	1.58	cornichon homolog 4 (Drosophila)
Mtus1	11.5	18.2	1.57	mitochondrial tumor suppressor 1
Hmgn1	88.0	138.2	1.57	high mobility group nucleosomal binding domain 1
Msrb2	11.3	17.7	1.57	methionine sulfoxide reductase B2
Piezo1	14.9	23.5	1.57	piezo-type mechanosensitive ion channel component 1
Ltbp1	21.2	33.3	1.57	latent transforming growth factor beta binding protein 1
Gpcpd1	27.8	43.7	1.57	glycerophosphocholine phosphodiesterase GDE1 homolog (S. cerevisiae)
Ncoa1	15.6	24.4	1.57	nuclear receptor coactivator 1
Jhdm1d	49.9	78.1	1.57	jumonji C domain-containing histone demethylase 1 homolog D (S. cerevisiae)
Myo18a	19.3	30.2	1.57	myosin XVIIIA
Aaed1	12.8	20.0	1.57	AhpC/TSA antioxidant enzyme domain containing 1
Ptprf	20.4	31.9	1.56	protein tyrosine phosphatase, receptor type, F
Phf16	75.8	118.4	1.56	PHD finger protein 16
Bmpr1a	26.1	40.8	1.56	bone morphogenetic protein receptor, type 1A
Cmc2	12.4	19.4	1.56	COX assembly mitochondrial protein 2
Mdm4	18.1	28.2	1.56	transformed mouse 3T3 cell double minute 4
8430427H17Rik	14.1	22.0	1.56	RIKEN cDNA 8430427H17 gene
Atp5g3	174.7	272.6	1.56	ATP synthase, H+ transporting, mitochondrial F0 complex, subunit C3 (subunit 9)
Fam126a	37.1	57.8	1.56	family with sequence similarity 126, member A

Gene symbol	FPKM XY	FPKM XX/*Sry*	Fold change	Gene name
Arid1a	33.3	51.8	1.56	AT rich interactive domain 1A (SWI-like)
Thbs1	45.1	70.3	1.56	thrombospondin 1
4930506M07Rik	54.1	84.2	1.55	RIKEN cDNA 4930506M07 gene
Wasf1	32.0	49.7	1.55	WAS protein family, member 1
Pcyt1b	12.0	18.6	1.55	phosphate cytidylyltransferase 1, choline, beta isoform
Ccser2	18.7	29.1	1.55	coiled-coil serine rich 2
Rcn2	25.7	39.8	1.55	reticulocalbin 2
Rps27l	69.7	108.1	1.55	ribosomal protein S27-like
Stxbp6	12.3	19.0	1.55	syntaxin binding protein 6 (amisyn)
Lamp2	39.9	61.8	1.55	lysosomal-associated membrane protein 2
Usp31	15.8	24.5	1.55	ubiquitin specific peptidase 31
Nr4a1	36.0	55.5	1.54	nuclear receptor subfamily 4, group A, member 1
Cox7b	14.2	21.8	1.54	cytochrome c oxidase subunit VIIb
Atp2b1	25.1	38.7	1.54	ATPase, Ca++ transporting, plasma membrane 1
Msrb3	37.1	57.1	1.54	methionine sulfoxide reductase B3
Npnt	15.8	24.3	1.54	nephronectin
Edem2	33.8	52.0	1.54	ER degradation enhancer, mannosidase alpha-like 2
Tmem123	56.4	86.6	1.54	transmembrane protein 123
Mrpl36	28.7	44.0	1.53	mitochondrial ribosomal protein L36
Cercam	14.6	22.3	1.53	cerebral endothelial cell adhesion molecule
Txnip	61.7	94.5	1.53	thioredoxin interacting protein
Slc39a6	82.9	126.8	1.53	solute carrier family 39 (metal ion transporter), member 6
Pbrm1	12.1	18.6	1.53	polybromo 1
Ncoa7	19.0	29.1	1.53	nuclear receptor coactivator 7
Anxa5	50.4	76.9	1.53	annexin A5
Rasl11b	12.5	19.0	1.53	RAS-like, family 11, member B
Alcam	17.7	27.0	1.52	activated leukocyte cell adhesion molecule
Ubr1	10.3	15.7	1.52	ubiquitin protein ligase E3 component n-recognin 1
Erap1	9.6	14.7	1.52	endoplasmic reticulum aminopeptidase 1
Golim4	11.7	17.8	1.52	golgi integral membrane protein 4
Ocrl	15.2	23.1	1.52	oculocerebrorenal syndrome of Lowe
Cobll1	15.4	23.4	1.52	Cobl-like 1
Smim11	20.6	31.4	1.52	small integral membrane protein 11
5430435G22Rik	31.5	48.0	1.52	RIKEN cDNA 5430435G22 gene
Rras	13.3	20.2	1.52	Harvey rat sarcoma oncogene, subgroup R
Pcmtd1	14.4	21.9	1.52	protein-L-isoaspartate (D-aspartate) O-methyltransferase domain containing 1
Thra	55.4	84.2	1.52	thyroid hormone receptor alpha
Fggy	11.5	17.4	1.52	FGGY carbohydrate kinase domain containing
Usp3	13.1	19.8	1.52	ubiquitin specific peptidase 3
Atp9b	44.9	68.1	1.52	ATPase, class II, type 9B
Degs1	19.4	29.4	1.51	degenerative spermatocyte homolog 1 (Drosophila)
Ing2	16.6	25.1	1.51	inhibitor of growth family, member 2
Ankra2	23.8	35.9	1.51	ankyrin repeat, family A (RFXANK-like), 2
Ccdc160	37.0	55.9	1.51	coiled-coil domain containing 160
AW549877	11.3	17.0	1.51	expressed sequence AW549877
Foxj2	12.3	18.5	1.51	forkhead box J2
Tet3	16.0	24.2	1.51	tet methylcytosine dioxygenase 3
2410127L17Rik	22.4	33.9	1.51	RIKEN cDNA 2410127L17 gene
Sat1	34.6	52.3	1.51	spermidine/spermine N1-acetyl transferase 1
Sptan1	106.2	160.3	1.51	spectrin alpha, non-erythrocytic 1
Slc1a4	11.9	18.0	1.51	solute carrier family 1 (glutamate/neutral amino acid transporter), member 4
Ndufc2	20.9	31.6	1.51	NADH dehydrogenase (ubiquinone) 1, subcomplex unknown, 2
Mrpl33	31.0	46.7	1.51	mitochondrial ribosomal protein L33
Pfn2	24.4	36.7	1.50	profilin 2

Gene symbol	FPKM XY	FPKM XX/*Sry*	Fold change	Gene name
4933415F23Rik	6.7	0.0	0.00	RIKEN cDNA 4933415F23 gene
Slc22a16	5.6	0.0	0.00	solute carrier family 22 (organic cation transporter), member 16
Qrich2	9.2	0.0	0.00	glutamine rich 2
Cox8c	21.8	0.0	0.00	cytochrome c oxidase subunit VIIIc
1700029F12Rik	18.5	0.0	0.00	RIKEN cDNA 1700029F12 gene
4933406F09Rik	5.0	0.0	0.00	RIKEN cDNA 4933406F09 gene
1700108J01Rik	5.8	0.0	0.00	RIKEN cDNA 1700108J01 gene
March11	9.7	0.0	0.00	membrane-associated ring finger (C3HC4) 11
Acr	5.2	0.0	0.00	acrosin prepropeptide
Gtsf1	9.3	0.0	0.00	gametocyte specific factor 1
Rpl39l	12.2	0.0	0.00	ribosomal protein L39-like
BC051142	10.1	0.0	0.00	cDNA sequence BC051142
Gtf2a1l	6.6	0.0	0.00	general transcription factor IIA, 1-like
Morc2b	5.6	0.0	0.00	microrchidia 2B
Pgk2	6.6	0.0	0.00	phosphoglycerate kinase 2
1700008K24Rik	15.5	0.0	0.00	RIKEN cDNA 1700008K24 gene
Pabpc2	11.6	0.0	0.00	poly(A) binding protein, cytoplasmic 2
Cetn1	14.2	0.0	0.00	centrin 1
Adad1	5.1	0.0	0.00	adenosine deaminase domain containing 1 (testis specific)
1700029M20Rik	6.0	0.0	0.00	RIKEN cDNA 1700029M20 gene
Dmrtb1	15.1	0.0	0.00	DMRT-like family B with proline-rich C-terminal, 1
Piwil1	8.2	0.0	0.00	piwi-like RNA-mediated gene silencing 1
Cox7b2	10.2	0.0	0.00	cytochrome c oxidase subunit VIIb2
Pdcl2	9.9	0.0	0.00	phosducin-like 2
Gk2	13.2	0.0	0.00	glycerol kinase 2
Gm3925	24.1	0.0	0.00	predicted gene 3925
1700123L14Rik	17.8	0.0	0.00	nucleoporin 50 pseudogene
Lrrc23	5.8	0.0	0.00	leucine rich repeat containing 23
Pbp2	6.3	0.0	0.00	phosphatidylethanolamine binding protein 2
Tex101	30.2	0.0	0.00	testis expressed gene 101
Lypd4	14.8	0.0	0.00	Ly6/Plaur domain containing 4
Stk33	5.9	0.0	0.00	serine/threonine kinase 33
Ccdc113	7.5	0.0	0.00	coiled-coil domain containing 113
Dpep3	15.2	0.0	0.00	dipeptidase 3
Mael	27.1	0.0	0.00	maelstrom homolog (Drosophila)
Ddx3y	11.9	0.0	0.00	DEAD (Asp-Glu-Ala-Asp) box polypeptide 3, Y-linked
Stag3	11.6	0.0	0.00	stromal antigen 3
Adam3	10.5	0.0	0.00	a disintegrin and metallopeptidase domain 3 (cyritestin)
Ccdc60	10.3	0.0	0.00	coiled-coil domain containing 60
Adam2	9.1	0.0	0.00	a disintegrin and metallopeptidase domain 2
Tuba3b	73.5	0.1	0.00	tubulin, alpha 3B
Eif2s3y	16.1	0.0	0.00	eukaryotic translation initiation factor 2, subunit 3, structural gene Y-linked
Clgn	15.0	0.0	0.00	calmegin
Crisp2	21.7	0.0	0.00	cysteine-rich secretory protein 2
Ppp3r2	6.9	0.0	0.00	protein phosphatase 3, regulatory subunit B, alpha isoform (calcineurin B, type II)
1700003M02Rik	12.9	0.0	0.00	RIKEN cDNA 1700003M02 gene
Tuba3a	77.0	0.1	0.00	tubulin, alpha 3A
Mroh2b	5.9	0.0	0.00	maestro heat-like repeat family member 2B
Spata4	21.2	0.0	0.00	spermatogenesis associated 4
Pom121l2	5.3	0.0	0.00	POM121 membrane glycoprotein-like 2 (rat)
Mroh4	10.4	0.0	0.00	maestro heat-like repeat family member 4
Fbp1	14.5	0.0	0.00	fructose bisphosphatase 1
Hrasls5	18.4	0.0	0.00	HRAS-like suppressor family, member 5

Gene symbol	FPKM XY	FPKM XX/*Sry*	Fold change	Gene name
Spata16	9.1	0.0	0.00	spermatogenesis associated 16
Ldhc	94.7	0.2	0.00	lactate dehydrogenase C
Gykl1	7.4	0.0	0.00	glycerol kinase-like 1
Tex40	17.4	0.1	0.00	testis expressed 40
4922502D21Rik	17.1	0.1	0.00	RIKEN cDNA 4922502D21 gene
Fabp9	40.4	0.1	0.00	fatty acid binding protein 9, testis
Hdgfl1	6.4	0.0	0.00	hepatoma derived growth factor-like 1
Dkkl1	27.8	0.1	0.00	dickkopf-like 1
Dnahc8	11.8	0.0	0.00	dynein, axonemal, heavy chain 8
Syngr4	11.6	0.0	0.00	synaptogyrin 4
Tekt1	8.5	0.0	0.00	tektin 1
Ddx25	14.0	0.1	0.00	DEAD (Asp-Glu-Ala-Asp) box polypeptide 25
Gsg2	8.3	0.0	0.00	germ cell-specific gene 2
Rsph6a	5.2	0.0	0.00	radial spoke head 6 homolog A (Chlamydomonas)
CK137956	5.1	0.0	0.00	cDNA sequence CK137956
Kctd19	9.6	0.0	0.00	potassium channel tetramerisation domain containing 19
4930544G11Rik	6.7	0.0	0.00	RIKEN cDNA 4930544G11 gene
4933416C03Rik	5.9	0.0	0.01	RIKEN cDNA 4933416C03 gene
Speer1-ps1	5.9	0.0	0.01	spermatogenesis associated glutamate (E)-rich protein 1, pseudogene 1
Papolb	5.8	0.0	0.01	poly (A) polymerase beta (testis specific)
Smc1b	5.7	0.0	0.01	structural maintenance of chromosomes 1B
Prss39	5.7	0.0	0.01	protease, serine, 39
1700010B08Rik	7.2	0.0	0.01	RIKEN cDNA 1700010B08 gene
Tcp11	23.0	0.1	0.01	t-complex protein 11
Zmynd10	12.3	0.1	0.01	zinc finger, MYND domain containing 10
4930511M11Rik	5.2	0.0	0.01	RIKEN cDNA 4930511M11 gene
Fam178b	8.2	0.1	0.01	family with sequence similarity 178, member B
Tcp10b	8.1	0.1	0.01	t-complex protein 10b
Ccdc136	15.9	0.1	0.01	coiled-coil domain containing 136
Syce1	12.5	0.1	0.01	synaptonemal complex central element protein 1
Ddx4	12.4	0.1	0.01	DEAD (Asp-Glu-Ala-Asp) box polypeptide 4
Spag6	9.2	0.1	0.01	sperm associated antigen 6
Adam5	14.5	0.1	0.01	a disintegrin and metallopeptidase domain 5
Ybx2	28.6	0.2	0.01	Y box protein 2
Cmtm2b	12.7	0.1	0.01	CKLF-like MARVEL transmembrane domain containing 2B
Clstn3	17.4	0.1	0.01	calsyntenin 3
1700011E24Rik	11.6	0.1	0.01	RIKEN cDNA 1700011E24 gene
1700029P11Rik	5.2	0.0	0.01	RIKEN cDNA 1700029P11 gene
Hey1	6.0	0.1	0.01	hairy/enhancer-of-split related with YRPW motif 1
1700001J11Rik	9.9	0.1	0.01	ring finger protein 19A pseudogene
Nek2	8.2	0.1	0.01	NIMA (never in mitosis gene a)-related expressed kinase 2
Gm3448,Tcte3	50.5	0.5	0.01	t-complex-associated testis expressed 3 | predicted gene 3448
Dbil5	6.9	0.1	0.01	diazepam binding inhibitor-like 5
40611(March10)	9.5	0.1	0.01	membrane-associated ring finger (C3HC4) 10
Gm7444	5.6	0.1	0.01	ring finger protein 19A pseudogene
Slc2a5	10.1	0.1	0.01	solute carrier family 2 (facilitated glucose transporter), member 5
Rpl10l	7.3	0.1	0.01	ribosomal protein L10-like
4732415M23Rik	14.5	0.2	0.01	RIKEN cDNA 4732415M23 gene
Cyct	5.4	0.1	0.01	cytochrome c, testis
Zpbp2	8.0	0.1	0.01	zona pellucida binding protein 2
Cmtm2a	16.6	0.2	0.01	CKLF-like MARVEL transmembrane domain containing 2A
Ldhal6b	20.0	0.2	0.01	lactate dehydrogenase A-like 6B
Tdrd6	7.3	0.1	0.01	tudor domain containing 6
Ccdc92	8.4	0.1	0.01	coiled-coil domain containing 92

Gene symbol	FPKM XY	FPKM XX/*Sry*	Fold change	Gene name
Tcp10c	5.3	0.1	0.02	t-complex protein 10c
Cetn4	7.0	0.1	0.02	centrin 4
Prps1l1	5.7	0.1	0.02	phosphoribosyl pyrophosphate synthetase 1-like 1
Shcbp1l	8.3	0.1	0.02	Shc SH2-domain binding protein 1-like
1700003E16Rik	5.7	0.1	0.02	RIKEN cDNA 1700003E16 gene
Prnd	15.9	0.3	0.02	prion protein dublet
Cdkn3	5.6	0.1	0.02	cyclin-dependent kinase inhibitor 3
Pabpc6	8.3	0.2	0.02	poly(A) binding protein, cytoplasmic 6
Ccdc38	6.8	0.1	0.02	coiled-coil domain containing 38
Ly6k	7.8	0.2	0.02	lymphocyte antigen 6 complex, locus K
Gm128	7.7	0.2	0.02	predicted gene 128
Tcfl5	5.7	0.1	0.02	transcription factor-like 5 (basic helix-loop-helix)
Adam32	9.1	0.2	0.02	a disintegrin and metallopeptidase domain 32
Eid3	10.4	0.2	0.02	EP300 interacting inhibitor of differentiation 3
Atp8b3	23.0	0.6	0.03	ATPase, class I, type 8B, member 3
Hs6st2	26.7	0.7	0.03	heparan sulfate 6-O-sulfotransferase 2
Fhl4	24.7	0.7	0.03	four and a half LIM domains 4
Ggn	7.2	0.2	0.03	gametogenetin
Ube2d2b	5.9	0.2	0.03	ubiquitin-conjugating enzyme E2D 2B
Alas2	19.1	0.6	0.03	aminolevulinic acid synthase 2, erythroid
Tcam1	6.4	0.2	0.03	testicular cell adhesion molecule 1
Sord	24.0	0.8	0.03	sorbitol dehydrogenase
Smpd5	5.5	0.2	0.03	sphingomyelin phosphodiesterase 5
Morn5	12.6	0.5	0.04	MORN repeat containing 5
Gsta2	126.9	4.7	0.04	glutathione S-transferase, alpha 2 (Yc2)
Atp2b3	5.3	0.2	0.04	ATPase, Ca++ transporting, plasma membrane 3
Crygc	7.1	0.3	0.04	crystallin, gamma C
Pacrg	7.1	0.3	0.04	PARK2 co-regulated
Tcp10a	6.2	0.3	0.04	t-complex protein 10a
Pcbp3	9.0	0.4	0.04	poly(rC) binding protein 3
Taf7l	5.5	0.2	0.04	TAF7-like RNA polymerase II, TATA box binding protein (TBP)-associated factor
Chga	8.3	0.4	0.04	chromogranin A
Dpm3	7.0	0.3	0.04	dolichyl-phosphate mannosyltransferase polypeptide 3
Mtl5	9.7	0.4	0.05	metallothionein-like 5, testis-specific (tesmin)
U90926	11.5	0.5	0.05	cDNA sequence U90926
Cox6b2	22.9	1.1	0.05	cytochrome c oxidase subunit VIb polypeptide 2
Spink2	21.5	1.1	0.05	serine peptidase inhibitor, Kazal type 2
Tnfaip2	14.4	0.7	0.05	tumor necrosis factor, alpha-induced protein 2
Adcy2	5.9	0.3	0.06	adenylate cyclase 2
1700019B21Rik	63.7	3.6	0.06	RIKEN cDNA 1700019B21 gene
Sycp3	10.4	0.6	0.06	synaptonemal complex protein 3
Cdc20	8.7	0.5	0.06	cell division cycle 20
Pgam2	28.9	1.8	0.06	phosphoglycerate mutase 2
Dnajb3	7.3	0.5	0.06	DnaJ (Hsp40) homolog, subfamily B, member 3
Ano1	41.5	2.6	0.06	anoctamin 1, calcium activated chloride channel
Hspb9	13.7	0.9	0.06	heat shock protein, alpha-crystallin-related, B9
1700028J19Rik	17.6	1.2	0.07	RIKEN cDNA 1700028J19 gene
Rasl2-9	6.0	0.4	0.07	RAS-like, family 2, locus 9
Ces2g	20.8	1.4	0.07	carboxylesterase 2G
Rfx2	6.0	0.4	0.07	regulatory factor X, 2 (influences HLA class II expression)
Tsnaxip1	5.4	0.4	0.07	translin-associated factor X (Tsnax) interacting protein 1
Cdca3	6.9	0.5	0.07	cell division cycle associated 3
Fam98c	8.7	0.6	0.07	family with sequence similarity 98, member C
Phf7	38.8	2.9	0.08	PHD finger protein 7

Gene symbol	FPKM XY	FPKM XX/*Sry*	Fold change	Gene name
Ropn1l	26.3	2.0	0.08	ropporin 1-like
Lrrc46	27.3	2.2	0.08	leucine rich repeat containing 46
Top2a	8.6	0.7	0.08	topoisomerase (DNA) II alpha
Hmga1	7.7	0.6	0.08	high mobility group AT-hook 1
Ttc30a2	6.0	0.5	0.09	tetratricopeptide repeat domain 30A2
Ccdc176	6.5	0.6	0.09	coiled-coil domain containing 176
Wnt6	7.5	0.7	0.09	wingless-related MMTV integration site 6
1700020D05Rik	8.6	0.8	0.09	RIKEN cDNA 1700020D05 gene
Anxa13	7.9	0.8	0.10	annexin A13
Gm4980	81.9	8.2	0.10	predicted gene 4980
6430548M08Rik	10.0	1.0	0.10	RIKEN cDNA 6430548M08 gene
Ptgds	15.3	1.6	0.10	prostaglandin D2 synthase (brain)
Mical2	19.2	2.0	0.10	microtubule associated monoxygenase, calponin and LIM domain containing 2
2210404O07Rik	47.6	5.0	0.10	RIKEN cDNA 2210404O07 gene
Ldlr	16.2	1.7	0.10	low density lipoprotein receptor
Asf1b	7.6	0.8	0.11	ASF1 anti-silencing function 1 homolog B (S. cerevisiae)
Fam229b	29.3	3.2	0.11	family with sequence similarity 229, member B
Acot7	20.3	2.3	0.11	acyl-CoA thioesterase 7
Aldh1a2	51.5	5.8	0.11	aldehyde dehydrogenase family 1, subfamily A2
Fyb	10.0	1.1	0.11	FYN binding protein
Aqp8	76.1	8.7	0.11	aquaporin 8
Ccdc65	10.3	1.3	0.12	coiled-coil domain containing 65
1700010I14Rik	18.6	2.3	0.12	RIKEN cDNA 1700010I14 gene
Hpca	5.7	0.7	0.12	hippocalcin
Dnaaf1	19.8	2.4	0.12	dynein, axonemal assembly factor 1
Cdca8	7.0	0.9	0.13	cell division cycle associated 8
Pebp4	7.9	1.0	0.13	phosphatidylethanolamine binding protein 4
Cdkn1a	24.6	3.2	0.13	cyclin-dependent kinase inhibitor 1A (P21)
Myh13	8.5	1.1	0.13	myosin, heavy polypeptide 13, skeletal muscle
Oit3	20.6	2.7	0.13	oncoprotein induced transcript 3
Dock6	9.0	1.2	0.14	dedicator of cytokinesis 6
Prss50	7.6	1.0	0.14	protease, serine, 50
AA467197	17.5	2.6	0.15	expressed sequence AA467197
Dhcr24	20.9	3.1	0.15	24-dehydrocholesterol reductase
Myl6b	71.2	10.9	0.15	myosin, light polypeptide 6B
Nid1	45.0	7.1	0.16	nidogen 1
Uba1y	8.0	1.3	0.17	ubiquitin-activating enzyme, Chr Y
Myh1	8.6	1.4	0.17	myosin, heavy polypeptide 1, skeletal muscle, adult
Stom	43.3	7.2	0.17	stomatin
Stmn1	229.7	39.6	0.17	stathmin 1
Sepw1	8.5	1.5	0.17	selenoprotein W, muscle 1
Slc15a1	6.6	1.1	0.17	solute carrier family 15 (oligopeptide transporter), member 1
Plk2	54.6	9.5	0.17	polo-like kinase 2
Rin2	9.8	1.7	0.18	Ras and Rab interactor 2
Gm1141	9.4	1.7	0.18	predicted gene 1141
Acrbp	12.9	2.4	0.18	proacrosin binding protein
Cabp1	20.4	3.8	0.18	calcium binding protein 1
Hspa2	52.0	9.7	0.19	heat shock protein 2
Aurka	9.4	1.8	0.19	aurora kinase A
Kif9	9.9	1.9	0.19	kinesin family member 9
Atp1a2	48.2	9.2	0.19	ATPase, Na+/K+ transporting, alpha 2 polypeptide
Lyar	32.4	6.3	0.19	Ly1 antibody reactive clone
Ypel1	9.4	1.8	0.19	yippee-like 1 (Drosophila)
Serpina3a	15.9	3.1	0.20	serine (or cysteine) peptidase inhibitor, clade A, member 3A

Gene symbol	FPKM XY	FPKM XX/*Sry*	Fold change	Gene name
Ccdc30	10.6	2.2	0.20	coiled-coil domain containing 30
Prelp	60.5	12.3	0.20	proline arginine-rich end leucine-rich repeat
Psmc3ip	6.5	1.3	0.20	proteasome (prosome, macropain) 26S subunit, ATPase 3, interacting protein
Sqle	7.5	1.6	0.21	squalene epoxidase
Acyp1	16.2	3.5	0.21	acylphosphatase 1, erythrocyte (common) type
Slc16a3	48.9	10.5	0.21	solute carrier family 16 (monocarboxylic acid transporters), member 3
Rgcc	10.1	2.2	0.22	regulator of cell cycle
Nptx2	26.5	5.7	0.22	neuronal pentraxin 2
Insig1	11.0	2.4	0.22	insulin induced gene 1
Slco1a5	14.6	3.2	0.22	solute carrier organic anion transporter family, member 1a5
Rab3il1	11.9	2.6	0.22	RAB3A interacting protein (rabin3)-like 1
Slc13a2	9.6	2.1	0.22	solute carrier family 13 (sodium-dependent dicarboxylate transporter), member 2
Gm648	69.7	15.5	0.22	predicted gene 648
Limd2	7.8	1.7	0.22	LIM domain containing 2
Ggnbp1	23.3	5.2	0.22	gametogenetin binding protein 1
Rnf32	13.7	3.1	0.22	ring finger protein 32
Gm11837	7.9	1.8	0.23	predicted gene 11837
Mrc1	35.3	8.3	0.23	mannose receptor, C type 1
Phkg2	31.6	7.5	0.24	phosphorylase kinase, gamma 2 (testis)
H2-DMa	8.3	2.0	0.24	histocompatibility 2, class II, locus DMa
1700039E15Rik	7.1	1.7	0.24	RIKEN cDNA 1700039E15 gene
Coprs	11.0	2.6	0.24	coordinator of PRMT5, differentiation stimulator
Fank1	7.4	1.8	0.24	fibronectin type 3 and ankyrin repeat domains 1
Eppin	284.8	72.2	0.25	epididymal peptidase inhibitor
Maged2	258.2	66.2	0.26	melanoma antigen, family D, 2
Entpd2	20.8	5.4	0.26	ectonucleoside triphosphate diphosphohydrolase 2
Tprn	8.3	2.2	0.26	taperin
Angel1	8.3	2.2	0.26	angel homolog 1 (Drosophila)
Ubash3a	8.0	2.1	0.26	ubiquitin associated and SH3 domain containing, A
Hdc	106.0	28.0	0.26	histidine decarboxylase
Lrrc27	11.4	3.0	0.27	leucine rich repeat containing 27
Actn3	32.7	8.9	0.27	actinin alpha 3
Usp1	9.3	2.5	0.27	ubiquitin specific peptidase 1
Hs3st1	7.0	1.9	0.27	heparan sulfate (glucosamine) 3-O-sulfotransferase 1
Tsacc	8.1	2.2	0.27	TSSK6 activating co-chaperone
Mvd	10.9	3.0	0.28	mevalonate (diphospho) decarboxylase
Arrb1	15.8	4.4	0.28	arrestin, beta 1
Lrrc4	11.4	3.2	0.28	leucine rich repeat containing 4
Syce3	8.2	2.3	0.28	synaptonemal complex central element protein 3
Tmem97	42.0	11.7	0.28	transmembrane protein 97
Dmrtc1b	72.8	20.7	0.28	DMRT-like family C1b
Ppp1r3c	20.3	5.8	0.29	protein phosphatase 1, regulatory (inhibitor) subunit 3C
Pcsk6	40.1	11.6	0.29	proprotein convertase subtilisin/kexin type 6
Prr5	11.6	3.4	0.29	proline rich 5 (renal)
Akr1c13	10.9	3.2	0.29	aldo-keto reductase family 1, member C13
Efhc1	8.8	2.6	0.29	EF-hand domain (C-terminal) containing 1
Creg1	88.5	25.8	0.29	cellular repressor of E1A-stimulated genes 1
Cldn3	9.4	2.7	0.29	claudin 3
Bscl2	28.9	8.5	0.29	Bernardinelli-Seip congenital lipodystrophy 2 homolog (human)
Slc9a3r1	24.1	7.1	0.29	solute carrier family 9 (sodium/hydrogen exchanger), member 3 regulator 1
Bahd1	47.2	13.8	0.29	bromo adjacent homology domain containing 1

Gene symbol	FPKM XY	FPKM XX/*Sry*	Fold change	Gene name
Fam78a	15.3	4.5	0.29	family with sequence similarity 78, member A
Cyp51	8.8	2.6	0.30	cytochrome P450, family 51
Frmpd1	24.7	7.4	0.30	FERM and PDZ domain containing 1
Il1a	20.9	6.4	0.30	interleukin 1 alpha
Rpl36	30.4	9.3	0.30	ribosomal protein L36
Idi1	8.8	2.7	0.31	isopentenyl-diphosphate delta isomerase
Ndufaf3	18.6	5.7	0.31	NADH dehydrogenase (ubiquinone) 1 alpha subcomplex, assembly factor 3
Lrwd1	11.8	3.6	0.31	leucine-rich repeats and WD repeat domain containing 1
Mgp	57.1	17.9	0.31	matrix Gla protein
Islr2	9.5	3.0	0.31	immunoglobulin superfamily containing leucine-rich repeat 2
Cacng5	23.5	7.4	0.31	calcium channel, voltage-dependent, gamma subunit 5
Hmgcs2	47.1	14.8	0.31	3-hydroxy-3-methylglutaryl-Coenzyme A synthase 2
Itpka	20.8	6.6	0.32	inositol 1,4,5-trisphosphate 3-kinase A
Stra6	61.9	19.6	0.32	stimulated by retinoic acid gene 6
Bbox1	23.8	7.6	0.32	butyrobetaine (gamma), 2-oxoglutarate dioxygenase 1 (gamma-butyrobetaine hydroxylase)
Rsph1	95.2	30.6	0.32	radial spoke head 1 homolog (Chlamydomonas)
Oscp1	12.7	4.1	0.32	organic solute carrier partner 1
Mt2	224.6	72.6	0.32	metallothionein 2
Smtnl2	18.7	6.1	0.32	smoothelin-like 2
Pgbd5	13.3	4.3	0.33	piggyBac transposable element derived 5
Kank3	20.8	6.9	0.33	KN motif and ankyrin repeat domains 3
Gramd3	32.9	10.9	0.33	GRAM domain containing 3
Tceb2	106.8	35.4	0.33	transcription elongation factor B (SIII), polypeptide 2
2200002J24Rik	8.4	2.8	0.34	RIKEN cDNA 2200002J24 gene
4930479M11Rik	8.0	2.7	0.34	RIKEN cDNA 4930479M11 gene
Svs5	11.0	3.7	0.34	seminal vesicle secretory protein 5
Qsox1	114.0	38.6	0.34	quiescin Q6 sulfhydryl oxidase 1
Bspry	7.8	2.6	0.34	B-box and SPRY domain containing
Dazl	11.8	4.0	0.34	deleted in azoospermia-like
Pde10a	9.0	3.1	0.34	phosphodiesterase 10A
Cxcr7	56.5	19.3	0.34	chemokine (C-X-C motif) receptor 7
Hsd17b3	11.5	3.9	0.34	hydroxysteroid (17-beta) dehydrogenase 3
Rangrf	29.1	10.0	0.34	RAN guanine nucleotide release factor
Pnpla6	27.0	9.3	0.34	patatin-like phospholipase domain containing 6
Rec8	66.1	22.7	0.34	REC8 homolog (yeast)
Dkk3	13.9	4.8	0.34	dickkopf homolog 3 (Xenopus laevis)
Myo1d	24.2	8.4	0.35	myosin ID
B4galt7	37.7	13.0	0.35	xylosylprotein beta1,4-galactosyltransferase, polypeptide 7 (galactosyltransferase I)
Haus4	12.5	4.3	0.35	HAUS augmin-like complex, subunit 4
Gm5617	8.0	2.8	0.35	predicted gene 5617
Slc16a4	17.0	5.9	0.35	solute carrier family 16 (monocarboxylic acid transporters), member 4
Tdrd1	24.7	8.6	0.35	tudor domain containing 1
1700026D08Rik	7.8	2.7	0.35	RIKEN cDNA 1700026D08 gene
Derl3	176.9	62.0	0.35	Der1-like domain family, member 3
Prph2	13.0	4.6	0.35	peripherin 2
Tox2	21.9	7.8	0.35	TOX high mobility group box family member 2
AU021092	31.2	11.0	0.35	expressed sequence AU021092
Vegfa	11.4	4.1	0.36	vascular endothelial growth factor A
Acsf2	35.4	12.6	0.36	acyl-CoA synthetase family member 2
Cchcr1	13.4	4.9	0.36	coiled-coil alpha-helical rod protein 1
Egr3	23.3	8.5	0.36	early growth response 3

Gene symbol	FPKM XY	FPKM XX/*Sry*	Fold change	Gene name
4930502E18Rik	41.5	15.2	0.37	RIKEN cDNA 4930502E18 gene
Mphosph8	16.3	5.9	0.37	M-phase phosphoprotein 8
Slc9a2	34.3	12.7	0.37	solute carrier family 9 (sodium/hydrogen exchanger), member 2
1700018G05Rik	335.1	124.3	0.37	RIKEN cDNA 1700018G05 gene
Cdkn1b	25.7	9.5	0.37	cyclin-dependent kinase inhibitor 1B
Ndrg1	48.8	18.1	0.37	N-myc downstream regulated gene 1
Aldh7a1	139.4	52.1	0.37	aldehyde dehydrogenase family 7, member A1
Wfdc1	250.9	93.9	0.37	WAP four-disulfide core domain 1
Fdps	34.7	13.0	0.37	farnesyl diphosphate synthetase
Tubb6	13.0	4.9	0.38	tubulin, beta 6 class V
Otop2	17.8	6.7	0.38	otopetrin 2
Serpini1	9.9	3.7	0.38	serine (or cysteine) peptidase inhibitor, clade I, member 1
Pdzd2	21.9	8.3	0.38	PDZ domain containing 2
Slc7a8	27.7	10.5	0.38	solute carrier family 7 (cationic amino acid transporter, y+ system), member 8
Ddt	33.2	12.6	0.38	D-dopachrome tautomerase
Krba1	13.8	5.3	0.38	KRAB-A domain containing 1
Hmox1	33.0	12.6	0.38	heme oxygenase (decycling) 1
Aldh9a1	50.9	19.5	0.38	aldehyde dehydrogenase 9, subfamily A1
Fkbpl	9.2	3.5	0.38	FK506 binding protein-like
Mpzl2	11.0	4.2	0.38	myelin protein zero-like 2
Ttc9	11.4	4.4	0.39	tetratricopeptide repeat domain 9
Egr2	35.6	13.9	0.39	early growth response 2
Ap1m2	8.2	3.2	0.39	adaptor protein complex AP-1, mu 2 subunit
Arhgef1	37.7	14.8	0.39	Rho guanine nucleotide exchange factor (GEF) 1
Lss	13.8	5.4	0.39	lanosterol synthase
Wdr35	8.8	3.5	0.39	WD repeat domain 35
Cdk20	12.7	5.0	0.39	cyclin-dependent kinase 20
Trf	51.1	20.1	0.39	transferrin
Piwil2	10.2	4.0	0.39	piwi-like RNA-mediated gene silencing 2
Isyna1	351.8	139.6	0.40	myo-inositol 1-phosphate synthase A1
Bend4	65.2	26.1	0.40	BEN domain containing 4
Fam132a	18.9	7.6	0.40	family with sequence similarity 132, member A
Il3ra	9.7	3.9	0.40	interleukin 3 receptor, alpha chain
Reep6	45.7	18.3	0.40	receptor accessory protein 6
Mum1	12.5	5.0	0.40	melanoma associated antigen (mutated) 1
Smg9	13.1	5.3	0.40	smg-9 homolog, nonsense mediated mRNA decay factor (C. elegans)
Tfeb	21.2	8.6	0.40	transcription factor EB
9630033F20Rik	33.4	13.5	0.41	RIKEN cDNA 9630033F20 gene
Pkig	29.9	12.1	0.41	protein kinase inhibitor, gamma
Arhgap22	12.1	4.9	0.41	Rho GTPase activating protein 22
Znhit2	9.5	3.9	0.41	zinc finger, HIT domain containing 2
Mroh1	8.8	3.6	0.41	maestro heat-like repeat family member 1
4930455F23Rik	16.5	6.7	0.41	RIKEN cDNA 4930455F23 gene
Mvk	11.3	4.6	0.41	mevalonate kinase
Pold4	9.1	3.7	0.41	polymerase (DNA-directed), delta 4
Sdk2	9.4	3.9	0.41	sidekick homolog 2 (chicken)
Kazn	22.8	9.4	0.41	kazrin, periplakin interacting protein
Rbm46	13.4	5.6	0.42	RNA binding motif protein 46
Smpdl3b	14.6	6.1	0.42	sphingomyelin phosphodiesterase, acid-like 3B
Josd2	10.6	4.4	0.42	Josephin domain containing 2
Morn2	17.9	7.5	0.42	MORN repeat containing 2
Rapgef3	13.1	5.5	0.42	Rap guanine nucleotide exchange factor (GEF) 3
Txnrd3	21.5	9.1	0.42	thioredoxin reductase 3

Gene symbol	FPKM XY	FPKM XX/*Sry*	Fold change	Gene name
Srebf2	36.4	15.4	0.42	sterol regulatory element binding factor 2
Emb	58.7	24.8	0.42	embigin
Atp2b4	52.5	22.2	0.42	ATPase, Ca++ transporting, plasma membrane 4
Igsf8	56.3	23.8	0.42	immunoglobulin superfamily, member 8
Slc10a6	8.9	3.8	0.42	solute carrier family 10 (sodium/bile acid cotransporter family), member 6
Fam134b	22.5	9.6	0.42	family with sequence similarity 134, member B
Tchp	10.2	4.4	0.42	trichoplein, keratin filament binding
Tmem180	64.7	27.5	0.43	transmembrane protein 180
Izumo4	9.2	3.9	0.43	IZUMO family member 4
Stxbp1	18.9	8.1	0.43	syntaxin binding protein 1
Il17re	36.1	15.4	0.43	interleukin 17 receptor E
C130074G19Rik	23.3	10.0	0.43	RIKEN cDNA C130074G19 gene
Aqp11	8.9	3.8	0.43	aquaporin 11
Fam110b	13.4	5.8	0.43	family with sequence similarity 110, member B
Setx	17.3	7.5	0.43	senataxin
Ank	35.9	15.6	0.43	progressive ankylosis
Enho	14.2	6.2	0.44	energy homeostasis associated
Slc39a13	12.8	5.6	0.44	solute carrier family 39 (metal ion transporter), member 13
Plekhf1	18.2	8.0	0.44	pleckstrin homology domain containing, family F (with FYVE domain) member 1
Gm16119	24.9	10.9	0.44	predicted gene 16119
Gm7120	15.6	6.9	0.44	predicted gene 7120
1700037H04Rik	33.2	14.7	0.44	RIKEN cDNA 1700037H04 gene
Dffa	13.0	5.7	0.44	DNA fragmentation factor, alpha subunit
Ppp1r15a	102.7	45.5	0.44	protein phosphatase 1, regulatory (inhibitor) subunit 15A
Junb	25.4	11.3	0.44	Jun-B oncogene
Sssca1	12.9	5.7	0.44	Sjogren s syndrome/scleroderma autoantigen 1 homolog (human)
Chchd6	33.8	15.0	0.44	coiled-coil-helix-coiled-coil-helix domain containing 6
S100a10	144.3	64.2	0.44	S100 calcium binding protein A10 (calpactin)
Mt1	275.8	122.7	0.44	metallothionein 1
Ssbp4	12.0	5.4	0.45	single stranded DNA binding protein 4
Zmiz2	34.4	15.3	0.45	zinc finger, MIZ-type containing 2
Pros1	49.8	22.2	0.45	protein S (alpha)
2810025M15Rik	47.2	21.1	0.45	RIKEN cDNA 2810025M15 gene
Tsx	322.9	144.5	0.45	testis specific X-linked gene
Slc2a8	13.3	6.0	0.45	solute carrier family 2, (facilitated glucose transporter), member 8
Lipg	71.1	31.9	0.45	lipase, endothelial
Npl	66.5	29.8	0.45	N-acetylneuraminate pyruvate lyase
Dixdc1	9.5	4.3	0.45	DIX domain containing 1
Slc30a1	26.5	12.0	0.45	solute carrier family 30 (zinc transporter), member 1
Fuz	10.7	4.8	0.45	fuzzy homolog (Drosophila)
Klf2	10.3	4.6	0.45	Kruppel-like factor 2 (lung)
Zfp35	11.8	5.4	0.45	zinc finger protein 35
Kctd11	9.5	4.3	0.45	potassium channel tetramerisation domain containing 11
Zfp280b	26.4	12.0	0.45	zinc finger protein 280B
Gm20605,Lrch4, Sap25	42.0	19.0	0.45	sin3 associated polypeptide | leucine-rich repeats and calponin homology (CH) domain containing 4 | predicted gene 20605
Mid1ip1	44.3	20.2	0.45	Mid1 interacting protein 1 (gastrulation specific G12-like (zebrafish))
Fbxl18	12.6	5.8	0.46	F-box and leucine-rich repeat protein 18
Dusp5	45.2	20.8	0.46	dual specificity phosphatase 5
Rasd1	116.7	53.8	0.46	RAS, dexamethasone-induced 1
Klhl36	53.1	24.5	0.46	kelch-like 36
Ift172	11.2	5.2	0.46	intraflagellar transport 172

Gene symbol	FPKM XY	FPKM XX/*Sry*	Fold change	Gene name
Agbl5	18.2	8.4	0.46	ATP/GTP binding protein-like 5
Man2b2	52.9	24.5	0.46	mannosidase 2, alpha B2
Mpnd	24.0	11.1	0.46	MPN domain containing
Spa17	17.2	8.0	0.46	sperm autoantigenic protein 17
Hsdl1	54.8	25.4	0.46	hydroxysteroid dehydrogenase like 1
Znhit1	19.8	9.2	0.46	zinc finger, HIT domain containing 1
Kctd12	28.7	13.4	0.47	potassium channel tetramerisation domain containing 12
Prkag1	35.9	16.8	0.47	protein kinase, AMP-activated, gamma 1 non-catalytic subunit
2610018G03Rik	9.8	4.6	0.47	RIKEN cDNA 2610018G03 gene
Id4	10.7	5.0	0.47	inhibitor of DNA binding 4
Pla2g6	41.8	19.7	0.47	phospholipase A2, group VI
Gm11744	66.5	31.3	0.47	predicted gene 11744
P2rx2	97.8	46.1	0.47	purinergic receptor P2X, ligand-gated ion channel, 2
Anxa4	11.6	5.5	0.47	annexin A4
Sash1	15.1	7.2	0.48	SAM and SH3 domain containing 1
Esrra	11.4	5.4	0.48	estrogen related receptor, alpha
Fchsd1	13.7	6.5	0.48	FCH and double SH3 domains 1
Tob2	12.3	5.9	0.48	transducer of ERBB2, 2
Calm2	314.3	150.5	0.48	calmodulin 2
Bik	24.8	11.9	0.48	BCL2-interacting killer
Pxmp4	13.1	6.3	0.48	peroxisomal membrane protein 4
Rdh11	14.7	7.0	0.48	retinol dehydrogenase 11
Zfp385a	25.8	12.5	0.48	zinc finger protein 385A
Nsdhl	17.2	8.4	0.49	NAD(P) dependent steroid dehydrogenase-like
Gpt2	62.7	30.4	0.49	glutamic pyruvate transaminase (alanine aminotransferase) 2
A730017C20Rik	45.9	22.3	0.49	RIKEN cDNA A730017C20 gene
Defb45	46.0	22.3	0.49	defensin beta 45
Tmub1	11.6	5.6	0.49	transmembrane and ubiquitin-like domain containing 1
Orai3	16.3	7.9	0.49	ORAI calcium release-activated calcium modulator 3
Phf1	10.0	4.9	0.49	PHD finger protein 1
Clybl	13.7	6.7	0.49	citrate lyase beta like
Arhgap31	35.2	17.3	0.49	Rho GTPase activating protein 31
Gpx1	40.0	19.7	0.49	glutathione peroxidase 1
Cxcr4	29.1	14.3	0.49	chemokine (C-X-C motif) receptor 4
Gem	10.0	4.9	0.49	GTP binding protein (gene overexpressed in skeletal muscle)
Pcyt2	17.6	8.8	0.50	phosphate cytidylyltransferase 2, ethanolamine
Cdk2ap2	43.3	21.7	0.50	CDK2-associated protein 2
Csda	48.6	24.3	0.50	cold shock domain protein A
Ints1	19.4	9.7	0.50	integrator complex subunit 1
Hes1	27.4	13.8	0.50	hairy and enhancer of split 1 (Drosophila)
Ppapdc3	11.2	5.6	0.50	phosphatidic acid phosphatase type 2 domain containing 3
Zcchc12	16.5	8.3	0.50	zinc finger, CCHC domain containing 12
Chpf	63.6	32.0	0.50	chondroitin polymerizing factor
Hcfc1r1	21.6	10.9	0.50	host cell factor C1 regulator 1 (XPO1-dependent)
Mcm7	14.6	7.3	0.50	minichromosome maintenance deficient 7 (S. cerevisiae)
Ccl27a	12.7	6.4	0.50	chemokine (C-C motif) ligand 27A
Akap12	290.8	146.6	0.50	A kinase (PRKA) anchor protein (gravin) 12
Ggct	14.7	7.4	0.50	gamma-glutamyl cyclotransferase
Cotl1	54.0	27.3	0.50	coactosin-like 1 (Dictyostelium)
Amhr2	1,156.3	583.8	0.50	anti-Mullerian hormone type 2 receptor
Mad2l2	14.9	7.5	0.51	MAD2 mitotic arrest deficient-like 2
Dis3l	13.1	6.6	0.51	DIS3 mitotic control homolog (S. cerevisiae)-like
Arl4d	106.0	53.6	0.51	ADP-ribosylation factor-like 4D
Marcksl1	462.0	233.7	0.51	MARCKS-like 1

Gene symbol	FPKM XY	FPKM XX/*Sry*	Fold change	Gene name
Aldh1a7	39.1	19.8	0.51	aldehyde dehydrogenase family 1, subfamily A7
2310047M10Rik	16.6	8.4	0.51	RIKEN cDNA 2310047M10 gene
Pcnt	18.5	9.4	0.51	pericentrin (kendrin)
Nckap5l	23.7	12.1	0.51	NCK-associated protein 5-like
Cdkn2d	66.6	34.0	0.51	cyclin-dependent kinase inhibitor 2D (p19, inhibits CDK4)
Espn	210.4	107.5	0.51	espin
Slc24a3	84.7	43.3	0.51	solute carrier family 24 (sodium/potassium/calcium exchanger), member 3
Rnf217	12.0	6.2	0.51	ring finger protein 217
Snx11	18.9	9.7	0.51	sorting nexin 11
Tpi1	29.2	15.0	0.51	triosephosphate isomerase 1
Entpd4	18.2	9.3	0.51	ectonucleoside triphosphate diphosphohydrolase 4
Entpd4	18.2	9.3	0.51	ectonucleoside triphosphate diphosphohydrolase 4
Meig1	44.2	22.8	0.52	meiosis expressed gene 1
Bloc1s4	19.4	10.0	0.52	biogenesis of organelles complex-1, subunit 4, cappuccino
Zc3hav1	13.8	7.1	0.52	zinc finger CCCH type, antiviral 1
BC055324	13.2	6.9	0.52	cDNA sequence BC055324
Camk2g	35.2	18.3	0.52	calcium/calmodulin-dependent protein kinase II gamma
Crat	15.8	8.2	0.52	carnitine acetyltransferase
Txk	12.4	6.5	0.52	TXK tyrosine kinase
Ivd	83.0	43.2	0.52	isovaleryl coenzyme A dehydrogenase
Med16	16.2	8.4	0.52	mediator complex subunit 16
Anxa9	28.4	14.9	0.52	annexin A9
Dcaf6	45.6	23.9	0.52	DDB1 and CUL4 associated factor 6
1600002K03Rik	23.6	12.4	0.53	RIKEN cDNA 1600002K03 gene
Tpx2	44.2	23.3	0.53	TPX2, microtubule-associated protein homolog (Xenopus laevis)
Stk19	14.0	7.4	0.53	serine/threonine kinase 19
Gps2	16.1	8.5	0.53	G protein pathway suppressor 2
Slc4a5	12.2	6.4	0.53	solute carrier family 4, sodium bicarbonate cotransporter, member 5
Emc10	47.0	24.8	0.53	ER membrane protein complex subunit 10
Mif	148.2	78.3	0.53	macrophage migration inhibitory factor
Tomm40l	21.5	11.4	0.53	translocase of outer mitochondrial membrane 40 homolog-like (yeast)
Rabac1	131.6	69.8	0.53	Rab acceptor 1 (prenylated)
Mob3a	18.4	9.8	0.53	MOB kinase activator 3A
Atp1b1	236.4	125.7	0.53	ATPase, Na+/K+ transporting, beta 1 polypeptide
Vps18	52.5	27.9	0.53	vacuolar protein sorting 18 (yeast)
Pitrm1	70.9	37.7	0.53	pitrilysin metallepetidase 1
Trim68	19.3	10.3	0.53	tripartite motif-containing 68
Atg2a	25.8	13.8	0.53	autophagy related 2A
Slc2a3	33.0	17.6	0.53	solute carrier family 2 (facilitated glucose transporter), member 3
H6pd	12.4	6.6	0.53	hexose-6-phosphate dehydrogenase (glucose 1-dehydrogenase)
Mea1	64.5	34.4	0.53	male enhanced antigen 1
Aldh4a1	15.9	8.5	0.53	aldehyde dehydrogenase 4 family, member A1
Phospho2	22.0	11.7	0.53	phosphatase, orphan 2
Ttll5	11.9	6.4	0.53	tubulin tyrosine ligase-like family, member 5
Klc4	31.3	16.8	0.54	kinesin light chain 4
Dnm3	12.5	6.7	0.54	dynamin 3
Dohh	25.2	13.5	0.54	deoxyhypusine hydroxylase/monooxygenase
Gnai1	31.0	16.6	0.54	guanine nucleotide binding protein (G protein), alpha inhibiting 1
Itm2c	273.1	147.6	0.54	integral membrane protein 2C
Ankrd54	58.7	31.9	0.54	ankyrin repeat domain 54
Ift140	30.5	16.6	0.54	intraflagellar transport 140
Tmem104	11.3	6.1	0.54	transmembrane protein 104

Gene symbol	FPKM XY	FPKM XX/*Sry*	Fold change	Gene name
Sulf1	63.4	34.5	0.54	sulfatase 1
Cln3	42.7	23.3	0.54	ceroid lipofuscinosis, neuronal 3, juvenile (Batten, Spielmeyer-Vogt disease)
Tarbp2	21.0	11.4	0.54	TAR (HIV) RNA binding protein 2
Pafah2	12.4	6.8	0.54	platelet-activating factor acetylhydrolase 2
Nucb2	14.1	7.7	0.55	nucleobindin 2
Rrp9	32.6	17.8	0.55	RRP9, small subunit (SSU) processome component, homolog (yeast)
Spata24	13.5	7.4	0.55	spermatogenesis associated 24
Polr2e	55.4	30.3	0.55	polymerase (RNA) II (DNA directed) polypeptide E
Fzd5	15.3	8.4	0.55	frizzled homolog 5 (Drosophila)
Rps29	399.9	219.3	0.55	ribosomal protein S29
St8sia2	62.8	34.5	0.55	ST8 alpha-N-acetyl-neuraminide alpha-2,8-sialyltransferase 2
Crnkl1	29.2	16.1	0.55	Crn, crooked neck-like 1 (Drosophila)
Nde1	17.8	9.8	0.55	nuclear distribution gene E homolog 1 (A nidulans)
1700045I19Rik	42.7	23.6	0.55	ring finger protein 138 pseudogene
Gm9112	356.5	196.6	0.55	predicted gene 9112
Wdr62	34.6	19.1	0.55	WD repeat domain 62
Morn4	17.6	9.7	0.55	MORN repeat containing 4
1810009A15Rik	40.8	22.6	0.55	RIKEN cDNA 1810009A15 gene
Fry	12.7	7.0	0.55	furry homolog (Drosophila)
1700011M02Rik	432.0	239.2	0.55	RIKEN cDNA 1700011M02 gene
Itgb5	132.3	73.2	0.55	integrin beta 5
Asnsd1	15.2	8.4	0.55	asparagine synthetase domain containing 1
Hmgcr	30.7	17.0	0.55	3-hydroxy-3-methylglutaryl-Coenzyme A reductase
Nop9	14.0	7.8	0.55	NOP9 nucleolar protein
Ncapd2	32.5	18.0	0.55	non-SMC condensin I complex, subunit D2
Irf9	17.3	9.6	0.56	interferon regulatory factor 9
Midn	38.8	21.6	0.56	midnolin
Myd88	12.4	6.9	0.56	myeloid differentiation primary response gene 88
Cryz	35.5	19.8	0.56	crystallin, zeta
Cks2	42.4	23.6	0.56	CDC28 protein kinase regulatory subunit 2
Ecsit	25.9	14.4	0.56	ECSIT homolog (Drosophila)
H2-K1	100.4	56.0	0.56	histocompatibility 2, K1, K region
Lsmd1	22.5	12.5	0.56	LSM domain containing 1
Ptms	125.2	69.9	0.56	parathymosin
Msto1	46.1	25.8	0.56	misato homolog 1 (Drosophila)
Snrnp25	15.4	8.6	0.56	small nuclear ribonucleoprotein 25 (U11/U12)
Hexim1	33.9	19.0	0.56	hexamethylene bis-acetamide inducible 1
Tom1l1	32.9	18.4	0.56	target of myb1-like 1 (chicken)
Cxcl1	61.2	34.4	0.56	chemokine (C-X-C motif) ligand 1
Dctpp1	19.0	10.7	0.56	dCTP pyrophosphatase 1
Prrg2	12.7	7.2	0.56	proline-rich Gla (G-carboxyglutamic acid) polypeptide 2
Cst9	1,805.6	1,015.6	0.56	cystatin 9
Gadd45g	76.9	43.3	0.56	growth arrest and DNA-damage-inducible 45 gamma
Ccdc85c	12.9	7.3	0.56	coiled-coil domain containing 85C
Slx4	11.8	6.6	0.56	SLX4 structure-specific endonuclease subunit homolog (S. cerevisiae)
Sf3b5	26.9	15.2	0.56	splicing factor 3b, subunit 5
Rsph3a	11.6	6.6	0.56	radial spoke 3A homolog (Chlamydomonas)
Dolpp1	16.3	9.2	0.56	dolichyl pyrophosphate phosphatase 1
Polr2i	43.1	24.4	0.57	polymerase (RNA) II (DNA directed) polypeptide I
Igf2r	19.6	11.1	0.57	insulin-like growth factor 2 receptor
Larp7	25.2	14.3	0.57	La ribonucleoprotein domain family, member 7
Ier2	64.8	36.9	0.57	immediate early response 2

Gene symbol	FPKM XY	FPKM XX/*Sry*	Fold change	Gene name
Ddx49	18.9	10.7	0.57	DEAD (Asp-Glu-Ala-Asp) box polypeptide 49
Exosc5	76.4	43.4	0.57	exosome component 5
Cdkn2b	20.8	11.8	0.57	cyclin-dependent kinase inhibitor 2B (p15, inhibits CDK4)
Tm6sf1	16.7	9.5	0.57	transmembrane 6 superfamily member 1
Gcc1	28.6	16.3	0.57	golgi coiled coil 1
Pld3	132.7	75.6	0.57	phospholipase D family, member 3
Cdc42ep4	36.4	20.8	0.57	CDC42 effector protein (Rho GTPase binding) 4
Mtap7d3	22.8	13.0	0.57	MAP7 domain containing 3
Dmrtc1a	260.0	148.3	0.57	DMRT-like family C1a
Slc52a2	13.0	7.4	0.57	solute carrier protein 52, member 2
Noa1	11.8	6.7	0.57	nitric oxide associated 1
Ppp1r11	17.5	10.0	0.57	protein phosphatase 1, regulatory (inhibitor) subunit 11
Pcna	58.9	33.7	0.57	proliferating cell nuclear antigen
Rabep2	22.1	12.7	0.57	rabaptin, RAB GTPase binding effector protein 2
Ndufs7	16.1	9.3	0.57	NADH dehydrogenase (ubiquinone) Fe-S protein 7
Skiv2l	19.7	11.3	0.58	superkiller viralicidic activity 2-like (S. cerevisiae)
Gpr137	19.6	11.3	0.58	G protein-coupled receptor 137
Ttll12	57.6	33.2	0.58	tubulin tyrosine ligase-like family, member 12
L3mbtl2	12.8	7.4	0.58	l(3)mbt-like 2 (Drosophila)
Sugp2	22.1	12.7	0.58	SURP and G patch domain containing 2
Tubb4b	108.2	62.5	0.58	tubulin, beta 4B class IVB
Sowahd	15.3	8.8	0.58	sosondowah ankyrin repeat domain family member D
Ccnk	23.8	13.8	0.58	cyclin K
Csrnp1	78.1	45.5	0.58	cysteine-serine-rich nuclear protein 1
Tubg1	25.6	14.9	0.58	tubulin, gamma 1
Asb6	17.0	9.9	0.58	ankyrin repeat and SOCS box-containing 6
Sox8	57.4	33.5	0.58	SRY-box containing gene 8
Gstm5	78.0	45.6	0.58	glutathione S-transferase, mu 5
Tctex1d2	13.7	8.0	0.58	Tctex1 domain containing 2
Elof1	49.6	29.0	0.58	elongation factor 1 homolog (ELF1, S. cerevisiae)
Nr1h2	47.6	27.9	0.59	nuclear receptor subfamily 1, group H, member 2
Nat9	15.7	9.2	0.59	N-acetyltransferase 9 (GCN5-related, putative)
Dnajb1	51.2	30.0	0.59	DnaJ (Hsp40) homolog, subfamily B, member 1
Eif2b2	30.3	17.8	0.59	eukaryotic translation initiation factor 2B, subunit 2 beta
Tmem175	12.3	7.2	0.59	transmembrane protein 175
Katnb1	18.1	10.6	0.59	katanin p80 (WD40-containing) subunit B 1
Lypla2	18.5	10.9	0.59	lysophospholipase 2
Rassf1	22.5	13.2	0.59	Ras association (RalGDS/AF-6) domain family member 1
Zfp932	13.8	8.1	0.59	zinc finger protein 932
Scamp3	22.7	13.4	0.59	secretory carrier membrane protein 3
Polr2l	46.3	27.3	0.59	polymerase (RNA) II (DNA directed) polypeptide L
Acd	12.4	7.3	0.59	adrenocortical dysplasia
A430005L14Rik	12.8	7.6	0.59	RIKEN cDNA A430005L14 gene
Sox4	38.9	23.0	0.59	SRY-box containing gene 4
Dhx16	17.7	10.5	0.59	DEAH (Asp-Glu-Ala-His) box polypeptide 16
Gba2	51.3	30.4	0.59	glucosidase beta 2
Fbxo46	20.9	12.4	0.59	F-box protein 46
Reep4	17.2	10.2	0.59	receptor accessory protein 4
Usp27x	30.5	18.2	0.60	ubiquitin specific peptidase 27, X chromosome
Arhgef25	16.5	9.9	0.60	Rho guanine nucleotide exchange factor (GEF) 25
Camsap3	40.4	24.0	0.60	calmodulin regulated spectrin-associated protein family, member 3
Cep63	19.6	11.7	0.60	centrosomal protein 63
Gtf2h4	13.1	7.8	0.60	general transcription factor II H, polypeptide 4
Scaf1	18.8	11.2	0.60	SR-related CTD-associated factor 1

Gene symbol	FPKM XY	FPKM XX/*Sry*	Fold change	Gene name
Edem1	27.7	16.5	0.60	ER degradation enhancer, mannosidase alpha-like 1
Rilpl2	61.3	36.6	0.60	Rab interacting lysosomal protein-like 2
Rbm42	27.3	16.3	0.60	RNA binding motif protein 42
Pomt1	22.1	13.2	0.60	protein-O-mannosyltransferase 1
Fam118b	21.5	12.9	0.60	family with sequence similarity 118, member B
Car7	52.1	31.2	0.60	carbonic anhydrase 7
Nags	23.0	13.8	0.60	N-acetylglutamate synthase
Odf2	84.6	50.7	0.60	outer dense fiber of sperm tails 2
Plgrkt	17.6	10.6	0.60	plasminogen receptor, C-terminal lysine transmembrane protein
Lta4h	47.7	28.7	0.60	leukotriene A4 hydrolase
Plekhh2	12.6	7.6	0.60	pleckstrin homology domain containing, family H (with MyTH4 domain) member 2
Trim37	20.5	12.3	0.60	tripartite motif-containing 37
Zfp593	14.2	8.5	0.60	zinc finger protein 593
Ecd	29.0	17.5	0.60	ecdysoneless homolog (Drosophila)
2010320M18Rik	17.2	10.4	0.60	RIKEN cDNA 2010320M18 gene
Atox1	165.9	100.1	0.60	ATX1 (antioxidant protein 1) homolog 1 (yeast)
Shc4	44.8	27.0	0.60	SHC (Src homology 2 domain containing) family, member 4
Ruvbl2	29.8	18.0	0.60	RuvB-like protein 2
Gab1	28.8	17.4	0.60	growth factor receptor bound protein 2-associated protein 1
Mmp14	92.7	56.0	0.60	matrix metallopeptidase 14 (membrane-inserted)
Vsig10	82.8	50.0	0.60	V-set and immunoglobulin domain containing 10
Larp1b	52.9	32.0	0.61	La ribonucleoprotein domain family, member 1B
Polr3a	18.9	11.4	0.61	polymerase (RNA) III (DNA directed) polypeptide A
Sigmar1	14.5	8.8	0.61	sigma non-opioid intracellular receptor 1
Sde2	28.3	17.2	0.61	SDE2 telomere maintenance homolog (S. pombe)
Zfpm1	29.8	18.1	0.61	zinc finger protein, multitype 1
Gpx4	118.5	71.9	0.61	glutathione peroxidase 4
4833420G17Rik	27.0	16.4	0.61	RIKEN cDNA 4833420G17 gene
Hdac1	33.9	20.6	0.61	histone deacetylase 1
Brsk1	23.3	14.2	0.61	BR serine/threonine kinase 1
Ift122	25.6	15.6	0.61	intraflagellar transport 122
Mipep	16.7	10.2	0.61	mitochondrial intermediate peptidase
Wbp1	37.8	23.1	0.61	WW domain binding protein 1
Nbas	22.5	13.7	0.61	neuroblastoma amplified sequence
Nasp	36.3	22.2	0.61	nuclear autoantigenic sperm protein (histone-binding)
Krtcap2	24.9	15.2	0.61	keratinocyte associated protein 2
Rnf126	27.5	16.8	0.61	ring finger protein 126
Myl9	132.2	80.9	0.61	myosin, light polypeptide 9, regulatory
Dtnbp1	16.3	10.0	0.61	dystrobrevin binding protein 1
Tmem55b	41.3	25.3	0.61	transmembrane protein 55b
Plekha7	13.0	8.0	0.61	pleckstrin homology domain containing, family A member 7
Umps	21.6	13.2	0.61	uridine monophosphate synthetase
Sowahc	31.3	19.2	0.61	sosondowah ankyrin repeat domain family member C
Ephb1	17.8	11.0	0.61	Eph receptor B1
Csnk1g2	44.8	27.5	0.61	casein kinase 1, gamma 2
Dmwd	85.3	52.4	0.61	dystrophia myotonica-containing WD repeat motif
Ddit4	38.7	23.8	0.62	DNA-damage-inducible transcript 4
Gpaa1	25.9	16.0	0.62	GPI anchor attachment protein 1
Fam73b	14.7	9.1	0.62	family with sequence similarity 73, member B
Calr4	23.5	14.5	0.62	calreticulin 4
Slc39a11	34.8	21.5	0.62	solute carrier family 39 (metal ion transporter), member 11
Pacs1	26.1	16.1	0.62	phosphofurin acidic cluster sorting protein 1
Bloc1s5	14.8	9.1	0.62	biogenesis of organelles complex-1, subunit 5, muted
Mrps11	25.4	15.6	0.62	mitochondrial ribosomal protein S11

Gene symbol	FPKM XY	FPKM XX/*Sry*	Fold change	Gene name
Edf1	43.3	26.7	0.62	endothelial differentiation-related factor 1
Nr0b1	93.2	57.5	0.62	nuclear receptor subfamily 0, group B, member 1
Gpm6b	90.2	55.6	0.62	glycoprotein m6b
Men1	20.6	12.7	0.62	multiple endocrine neoplasia 1
Rplp1	256.9	158.5	0.62	ribosomal protein, large, P1
Sppl2b	24.8	15.3	0.62	signal peptide peptidase like 2B
Dnalc4	19.3	11.9	0.62	dynein, axonemal, light chain 4
Pop7	13.6	8.4	0.62	processing of precursor 7, ribonuclease P family, (S. cerevisiae)
Inpp5b	85.3	52.8	0.62	inositol polyphosphate-5-phosphatase B
Dak	22.4	13.9	0.62	dihydroxyacetone kinase 2 homolog (yeast)
Plp1	44.4	27.5	0.62	proteolipid protein (myelin) 1
Cdo1	53.7	33.3	0.62	cysteine dioxygenase 1, cytosolic
Cep89	17.2	10.7	0.62	centrosomal protein 89
Fbxo34	87.5	54.3	0.62	F-box protein 34
Noc4l	15.0	9.3	0.62	nucleolar complex associated 4 homolog (S. cerevisiae)
Abcf2	31.8	19.7	0.62	ATP-binding cassette, sub-family F (GCN20), member 2
Kndc1	42.5	26.4	0.62	kinase non-catalytic C-lobe domain (KIND) containing 1
Ldha	74.1	46.1	0.62	lactate dehydrogenase A
Ogdhl	33.7	21.0	0.62	oxoglutarate dehydrogenase-like
Pex14	39.0	24.3	0.62	peroxisomal biogenesis factor 14
Usp20	23.4	14.6	0.62	ubiquitin specific peptidase 20
Nomo1	88.2	55.1	0.62	nodal modulator 1
2010204K13Rik	33.0	20.6	0.62	RIKEN cDNA 2010204K13 gene
Sh3glb2	19.6	12.2	0.62	SH3-domain GRB2-like endophilin B2
Creld2	43.9	27.5	0.62	cysteine-rich with EGF-like domains 2
D2Wsu81e	27.9	17.5	0.63	DNA segment, Chr 2, Wayne State University 81, expressed
Dus3l	19.9	12.5	0.63	dihydrouridine synthase 3-like (S. cerevisiae)
Mns1	59.4	37.2	0.63	meiosis-specific nuclear structural protein 1
Slc41a2	63.8	40.0	0.63	solute carrier family 41, member 2
Fntb	23.5	14.8	0.63	farnesyltransferase, CAAX box, beta
Inhbb	108.2	67.9	0.63	inhibin beta-B
Hmox2	41.5	26.1	0.63	heme oxygenase (decycling) 2
Acad11	17.5	11.0	0.63	acyl-Coenzyme A dehydrogenase family, member 11
Gatad2a	48.6	30.6	0.63	GATA zinc finger domain containing 2A
Uchl1	151.3	95.4	0.63	ubiquitin carboxy-terminal hydrolase L1
Fam214b	34.1	21.5	0.63	family with sequence similarity 214, member B
Polr3k	14.9	9.4	0.63	polymerase (RNA) III (DNA directed) polypeptide K
Pla2g7	107.8	68.1	0.63	phospholipase A2, group VII (platelet-activating factor acetylhydrolase, plasma)
Zswim8	131.0	82.9	0.63	zinc finger SWIM-type containing 8
Taf4b	14.9	9.4	0.63	TAF4B RNA polymerase II, TATA box binding protein (TBP)-associated factor
Ehd1	43.4	27.5	0.63	EH-domain containing 1
Dnm2	46.4	29.4	0.63	dynamin 2
Idnk	23.3	14.7	0.63	idnK gluconokinase homolog (E. coli)
Mrpl52	34.8	22.0	0.63	mitochondrial ribosomal protein L52
Tbl3	26.5	16.8	0.63	transducin (beta)-like 3
Msrb1	23.4	14.8	0.63	methionine sulfoxide reductase B1
4933402E13Rik	254.2	161.2	0.63	RIKEN cDNA 4933402E13 gene
Pgp	40.7	25.9	0.64	phosphoglycolate phosphatase
Ap1m1	30.6	19.5	0.64	adaptor-related protein complex AP-1, mu subunit 1
Ppox	20.7	13.2	0.64	protoporphyrinogen oxidase
Polr2f	51.3	32.6	0.64	polymerase (RNA) II (DNA directed) polypeptide F
Synpo	21.8	13.9	0.64	synaptopodin
Baiap2	29.5	18.8	0.64	brain-specific angiogenesis inhibitor 1-associated protein 2

Gene symbol	FPKM XY	FPKM XX/*Sry*	Fold change	Gene name
Hspbp1	20.1	12.8	0.64	HSPA (heat shock 70kDa) binding protein, cytoplasmic cochaperone 1
Nxt1	16.0	10.2	0.64	NTF2-related export protein 1
Abat	106.0	67.6	0.64	4-aminobutyrate aminotransferase
Eif2ak1	45.5	29.1	0.64	eukaryotic translation initiation factor 2 alpha kinase 1
Mecr	18.8	12.0	0.64	mitochondrial trans-2-enoyl-CoA reductase
Ncor2	59.1	37.8	0.64	nuclear receptor co-repressor 2
Zfp36	161.5	103.4	0.64	zinc finger protein 36
Rnf40	29.9	19.1	0.64	ring finger protein 40
Psmc3	88.6	56.8	0.64	proteasome (prosome, macropain) 26S subunit, ATPase 3
Nol6	38.6	24.8	0.64	nucleolar protein family 6 (RNA-associated)
Mink1	26.9	17.3	0.64	misshapen-like kinase 1 (zebrafish)
Lsm2	17.5	11.3	0.64	LSM2 homolog, U6 small nuclear RNA associated (S. cerevisiae)
Ccdc104	32.3	20.7	0.64	coiled-coil domain containing 104
Ncrna00086	67.6	43.4	0.64	non-protein coding RNA 86
Ifi35	14.3	9.2	0.64	interferon-induced protein 35
Ptprv	113.1	72.8	0.64	protein tyrosine phosphatase, receptor type, V
Tcp1	115.0	74.1	0.64	t-complex protein 1
Ttc4	18.9	12.1	0.64	tetratricopeptide repeat domain 4
Mpi	60.2	38.8	0.64	mannose phosphate isomerase
Cbx4	20.4	13.1	0.64	chromobox 4
Ccdc9	15.4	9.9	0.64	coiled-coil domain containing 9
Sil1	49.9	32.2	0.64	endoplasmic reticulum chaperone SIL1 homolog (S. cerevisiae)
Dcbld1	32.1	20.7	0.65	discoidin, CUB and LCCL domain containing 1
Kpna2	44.8	28.9	0.65	karyopherin (importin) alpha 2
Mus81	15.9	10.3	0.65	MUS81 endonuclease homolog (yeast)
Thop1	19.2	12.4	0.65	thimet oligopeptidase 1
Ercc1	17.5	11.3	0.65	excision repair cross-complementing rodent repair deficiency, complementation group 1
Fam108a	60.7	39.3	0.65	family with sequence similarity 108, member A
Plec	37.4	24.2	0.65	plectin
Dpysl5	48.6	31.4	0.65	dihydropyrimidinase-like 5
Cgnl1	23.6	15.3	0.65	cingulin-like 1
Sphk2	63.7	41.3	0.65	sphingosine kinase 2
Mpv17l2	33.5	21.7	0.65	MPV17 mitochondrial membrane protein-like 2
G6pc3	91.9	59.6	0.65	glucose 6 phosphatase, catalytic, 3
Babam1	26.8	17.4	0.65	BRISC and BRCA1 A complex member 1
Rgl2	26.9	17.5	0.65	ral guanine nucleotide dissociation stimulator-like 2
Rfc2	17.5	11.3	0.65	replication factor C (activator 1) 2
Rnf31	16.4	10.7	0.65	ring finger protein 31
Lrpap1	209.8	136.4	0.65	low density lipoprotein receptor-related protein associated protein 1
Pkdcc	36.2	23.6	0.65	protein kinase domain containing, cytoplasmic
Ppp1r16a	25.8	16.8	0.65	protein phosphatase 1, regulatory (inhibitor) subunit 16A
Sfi1	17.0	11.1	0.65	Sfi1 homolog, spindle assembly associated (yeast)
Rpa1	20.0	13.0	0.65	replication protein A1
Map2k2	36.0	23.4	0.65	mitogen-activated protein kinase kinase 2
Agfg2	85.1	55.4	0.65	ArfGAP with FG repeats 2
Sc4mol	42.5	27.7	0.65	sterol-C4-methyl oxidase-like
Arl3	29.9	19.5	0.65	ADP-ribosylation factor-like 3
Tdp1	19.9	13.0	0.65	tyrosyl-DNA phosphodiesterase 1
Tex19.2	100.7	65.7	0.65	testis expressed gene 19.2
Tars2	41.5	27.1	0.65	threonyl-tRNA synthetase 2, mitochondrial (putative)
AA414768	37.7	24.6	0.65	expressed sequence AA414768
Zdhhc12	62.8	41.0	0.65	zinc finger, DHHC domain containing 12
Ints10	22.3	14.6	0.65	integrator complex subunit 10

Gene symbol	FPKM XY	FPKM XX/*Sry*	Fold change	Gene name
Lrrfip2	22.5	14.7	0.65	leucine rich repeat (in FLII) interacting protein 2
Rps15	212.0	138.7	0.65	ribosomal protein S15
Unc119b	39.6	25.9	0.65	unc-119 homolog B (C. elegans)
Gas2l1	170.3	111.5	0.65	growth arrest-specific 2 like 1
Chp2	178.3	117.0	0.66	calcineurin-like EF hand protein 2
Bet1l	20.3	13.3	0.66	blocked early in transport 1 homolog (S. cerevisiae)-like
Ipo4	38.4	25.2	0.66	importin 4
3010026O09Rik	19.6	12.9	0.66	RIKEN cDNA 3010026O09 gene
Tspan7	21.3	14.0	0.66	tetraspanin 7
Atp5sl	20.4	13.4	0.66	ATP5S-like
Aldh1b1	41.7	27.4	0.66	aldehyde dehydrogenase 1 family, member B1
Hectd3	17.7	11.7	0.66	HECT domain containing 3
Slc7a4	93.9	61.8	0.66	solute carrier family 7 (cationic amino acid transporter, y+ system), member 4
Irs2	65.9	43.4	0.66	insulin receptor substrate 2
Lmbr1l	60.5	39.9	0.66	limb region 1 like
Mogs	19.6	12.9	0.66	mannosyl-oligosaccharide glucosidase
Cog6	28.3	18.7	0.66	component of oligomeric golgi complex 6
Mbd6	19.7	13.0	0.66	methyl-CpG binding domain protein 6
BC017158	19.1	12.6	0.66	cDNA sequence BC017158
Eif4g3	53.2	35.1	0.66	eukaryotic translation initiation factor 4 gamma, 3
Lyplal1	21.2	14.0	0.66	lysophospholipase-like 1
Nf2	121.6	80.3	0.66	neurofibromatosis 2
Dbn1	15.1	10.0	0.66	drebrin 1
Mrpl55	19.7	13.0	0.66	mitochondrial ribosomal protein L55
Fbxl19	98.5	65.1	0.66	F-box and leucine-rich repeat protein 19
Kcnh2	58.9	38.9	0.66	potassium voltage-gated channel, subfamily H (eag-related), member 2
Ubac2	95.2	62.9	0.66	ubiquitin associated domain containing 2
Klf16	34.6	22.9	0.66	Kruppel-like factor 16
8030411F24Rik	234.9	155.4	0.66	RIKEN cDNA 8030411F24 gene
Eif2b4	27.4	18.1	0.66	eukaryotic translation initiation factor 2B, subunit 4 delta
Scrn2	59.4	39.3	0.66	secernin 2
Pi4k2b	21.9	14.5	0.66	phosphatidylinositol 4-kinase type 2 beta
Caskin1	53.2	35.3	0.66	CASK interacting protein 1
Ctxn1	15.3	10.2	0.66	cortexin 1
Atp11b	34.9	23.2	0.66	ATPase, class VI, type 11B
Plk1s1	23.4	15.6	0.66	polo-like kinase 1 substrate 1
Cherp	22.6	15.0	0.66	calcium homeostasis endoplasmic reticulum protein
Atp6v0a1	47.8	31.8	0.66	ATPase, H+ transporting, lysosomal V0 subunit A1
Neurl4	24.5	16.3	0.66	neuralized homolog 4 (Drosophila)
Tob1	42.1	28.0	0.66	transducer of ErbB-2.1
Tada3	22.4	14.9	0.66	transcriptional adaptor 3

Supplemental Table S6. GO and KEGG pathway analysis using genes up-regulated in XX/*Sry* Sertoli cells at P21

ID	Term	Fold enrichment	P-value
GO:0050840	extracellular matrix binding	13.05	1.1.E-05
GO:0005520	insulin-like growth factor binding	10.60	1.1.E-03
GO:0001755	neural crest cell migration	10.40	1.2.E-03
GO:0014032	neural crest cell development	8.70	5.6.E-04
GO:0014033	neural crest cell differentiation	8.70	5.6.E-04
GO:0048762	mesenchymal cell differentiation	6.84	5.1.E-04
GO:0060485	mesenchyme development	6.70	5.7.E-04
GO:0045785	positive regulation of cell adhesion	6.68	1.9.E-03
GO:0040017	positive regulation of locomotion	6.52	2.1.E-03
GO:0019838	growth factor binding	6.48	2.1.E-05
GO:0030335	positive regulation of cell migration	6.47	7.1.E-03
GO:0014031	mesenchymal cell development	6.11	2.8.E-03
GO:0030027	lamellipodium	6.06	3.3.E-04
GO:0030334	regulation of cell migration	5.20	1.2.E-04
GO:0031589	cell-substrate adhesion	5.04	6.5.E-03
GO:0040012	regulation of locomotion	4.78	9.8.E-05
GO:0030155	regulation of cell adhesion	4.58	7.6.E-04
GO:0051270	regulation of cell motion	4.47	3.9.E-04
GO:0051216	cartilage development	4.29	5.7.E-03
GO:0031252	cell leading edge	4.26	5.5.E-04
GO:0043405	regulation of MAP kinase activity	3.99	8.1.E-03
GO:0008201	heparin binding	3.93	8.6.E-03
GO:0060348	bone development	3.65	3.3.E-03
GO:0001503	ossification	3.61	6.7.E-03
GO:0048514	blood vessel morphogenesis	3.38	2.6.E-04
GO:0007169	transmembrane receptor protein tyrosine kinase signaling pathway	3.24	7.0.E-04
GO:0008509	anion transmembrane transporter activity	3.23	6.8.E-03
GO:0007167	enzyme linked receptor protein signaling pathway	3.15	6.0.E-05
GO:0001944	vasculature development	3.06	2.4.E-04
GO:0015629	actin cytoskeleton	3.03	1.2.E-03
GO:0001568	blood vessel development	2.94	6.1.E-04
GO:0035239	tube morphogenesis	2.80	9.6.E-03
GO:0001501	skeletal system development	2.69	9.5.E-04
GO:0051094	positive regulation of developmental process	2.68	5.3.E-03
GO:0044057	regulation of system process	2.62	9.6.E-03
GO:0035295	tube development	2.36	9.5.E-03
GO:0031012	extracellular matrix	2.32	5.4.E-03
GO:0007155	cell adhesion	2.30	1.2.E-04
GO:0022610	biological adhesion	2.30	1.2.E-04
GO:0045944	positive regulation of transcription from RNA polymerase II promoter	2.27	3.5.E-03
GO:0005578	proteinaceous extracellular matrix	2.25	9.7.E-03
GO:0008092	cytoskeletal protein binding	2.14	3.5.E-03
GO:0045893	positive regulation of transcription, DNA-dependent	2.07	6.4.E-03
GO:0051254	positive regulation of RNA metabolic process	2.06	6.9.E-03
GO:0042127	regulation of cell proliferation	2.05	2.0.E-03
GO:0006357	regulation of transcription from RNA polymerase II promoter	2.02	1.1.E-03
GO:0005509	calcium ion binding	2.00	1.1.E-04
GO:0044421	extracellular region part	1.85	1.6.E-03
GO:0005576	extracellular region	1.42	8.8.E-03
GO:0043167	ion binding	1.27	2.9.E-03
GO:0046872	metal ion binding	1.26	4.3.E-03
GO:0043169	cation binding	1.25	5.8.E-03
mmu04512	ECM-receptor interaction	4.32	2.3.E-03
mmu04510	Focal adhesion	2.94	1.3.E-03
mmu04810	Regulation of actin cytoskeleton	2.89	9.4.E-04

Supplemental Table S7.
GO and KEGG pathway analysis using genes down-regulated in XX/*Sry* Sertoli cells at P21

ID	Term	Fold enrichment	P-value
GO:0033391	chromatoid body	26.7	4.2.E-07
GO:0034587	piRNA metabolic process	22.0	4.5.E-04
GO:0004459	L-lactate dehydrogenase activity	20.2	7.8.E-03
GO:0043186	P granule	19.0	6.2.E-05
GO:0060293	germ plasm	19.0	6.2.E-05
GO:0045495	pole plasm	19.0	6.2.E-05
GO:0004029	aldehyde dehydrogenase (NAD) activity	13.5	2.5.E-03
GO:0030291	protein serine/threonine kinase inhibitor activity	12.0	3.6.E-03
GO:0006695	cholesterol biosynthetic process	12.0	5.3.E-08
GO:0005844	polysome	11.4	1.1.E-04
GO:0016126	sterol biosynthetic process	11.0	3.8.E-09
GO:0005665	DNA-directed RNA polymerase II, core complex	9.7	6.9.E-03
GO:0008299	isoprenoid biosynthetic process	8.8	1.0.E-04
GO:0007339	binding of sperm to zona pellucida	8.1	2.8.E-03
GO:0030374	ligand-dependent nuclear receptor transcription coactivator activity	7.7	8.5.E-04
GO:0035036	sperm-egg recognition	7.6	3.5.E-03
GO:0006367	transcription initiation from RNA polymerase II promoter	7.6	3.5.E-03
GO:0001673	male germ cell nucleus	7.4	3.9.E-03
GO:0009988	cell-cell recognition	6.9	5.2.E-03
GO:0000795	synaptonemal complex	6.7	5.8.E-03
GO:0043073	germ cell nucleus	6.1	8.3.E-03
GO:0007051	spindle organization	5.9	3.0.E-03
GO:0000794	condensed nuclear chromosome	5.7	1.4.E-04
GO:0016620	oxidoreductase activity, acting on the aldehyde or oxo group of donors, NAD or NADP as acceptor	5.6	3.9.E-03
GO:0051321	meiotic cell cycle	5.5	2.0.E-08
GO:0006694	steroid biosynthetic process	5.4	1.4.E-06
GO:0051327	M phase of meiotic cell cycle	5.3	9.2.E-08
GO:0007126	meiosis	5.3	9.2.E-08
GO:0044450	microtubule organizing center part	5.2	5.5.E-03
GO:0006720	isoprenoid metabolic process	5.1	3.4.E-04
GO:0016125	sterol metabolic process	5.0	3.5.E-06
GO:0030176	integral to endoplasmic reticulum membrane	4.8	7.2.E-03
GO:0035257	nuclear hormone receptor binding	4.8	7.8.E-03
GO:0008203	cholesterol metabolic process	4.7	4.0.E-05
GO:0007127	meiosis I	4.7	8.1.E-03
GO:0034062	RNA polymerase activity	4.6	8.8.E-03
GO:0003899	DNA-directed RNA polymerase activity	4.6	8.8.E-03
GO:0019887	protein kinase regulator activity	4.6	2.9.E-04
GO:0006366	transcription from RNA polymerase II promoter	4.5	6.8.E-05
GO:0006096	glycolysis	4.4	4.8.E-03
GO:0019861	flagellum	4.4	9.4.E-04
GO:0016591	DNA-directed RNA polymerase II, holoenzyme	4.3	1.1.E-03
GO:0006007	glucose catabolic process	4.2	2.6.E-03
GO:0019320	hexose catabolic process	4.2	2.6.E-03
GO:0046365	monosaccharide catabolic process	4.1	3.2.E-03
GO:0003729	mRNA binding	4.0	3.6.E-03
GO:0007283	spermatogenesis	4.0	2.2.E-12
GO:0048232	male gamete generation	4.0	2.2.E-12
GO:0007286	spermatid development	3.7	5.3.E-03
GO:0019207	kinase regulator activity	3.7	1.4.E-03
GO:0044275	cellular carbohydrate catabolic process	3.7	5.8.E-03
GO:0007281	germ cell development	3.5	2.9.E-04
GO:0048515	spermatid differentiation	3.5	7.6.E-03
GO:0001666	response to hypoxia	3.4	8.3.E-03
GO:0046164	alcohol catabolic process	3.4	9.0.E-03

GO:0070482	response to oxygen levels	3.4	9.0.E-03
GO:0048610	reproductive cellular process	3.3	4.7.E-06
GO:0007276	gamete generation	3.2	2.9.E-10
GO:0000279	M phase	3.2	1.1.E-08
GO:0048609	reproductive process in a multicellular organism	3.2	7.3.E-12
GO:0032504	multicellular organism reproduction	3.2	7.3.E-12
GO:0019953	sexual reproduction	3.1	5.0.E-11
GO:0006417	regulation of translation	3.0	3.4.E-03
GO:0022403	cell cycle phase	3.0	1.0.E-08
GO:0022402	cell cycle process	3.0	3.1.E-10
GO:0000793	condensed chromosome	3.0	2.2.E-03
GO:0000228	nuclear chromosome	2.9	1.9.E-03
GO:0010608	posttranscriptional regulation of gene expression	2.8	9.8.E-04
GO:0006006	glucose metabolic process	2.8	1.7.E-03
GO:0008202	steroid metabolic process	2.7	7.7.E-04
GO:0006351	transcription, DNA-dependent	2.7	4.8.E-03
GO:0005819	spindle	2.7	7.6.E-03
GO:0030005	cellular di-, tri-valent inorganic cation homeostasis	2.7	3.5.E-03
GO:0032774	RNA biosynthetic process	2.6	6.1.E-03
GO:0019318	hexose metabolic process	2.6	1.3.E-03
GO:0005815	microtubule organizing center	2.5	7.4.E-04
GO:0030003	cellular cation homeostasis	2.5	3.6.E-03
GO:0007049	cell cycle	2.5	3.8.E-10
GO:0003006	reproductive developmental process	2.5	9.5.E-05
GO:0044092	negative regulation of molecular function	2.5	8.6.E-03
GO:0005813	centrosome	2.5	2.7.E-03
GO:0055066	di-, tri-valent inorganic cation homeostasis	2.4	6.9.E-03
GO:0008610	lipid biosynthetic process	2.4	1.1.E-04
GO:0000278	mitotic cell cycle	2.4	5.9.E-04
GO:0042175	nuclear envelope-endoplasmic reticulum network	2.3	7.1.E-03
GO:0005996	monosaccharide metabolic process	2.3	4.1.E-03
GO:0055080	cation homeostasis	2.2	7.2.E-03
GO:0005874	microtubule	2.2	1.7.E-03
GO:0006873	cellular ion homeostasis	2.1	3.2.E-03
GO:0015630	microtubule cytoskeleton	2.1	7.8.E-05
GO:0055082	cellular chemical homeostasis	2.1	4.2.E-03
GO:0030529	ribonucleoprotein complex	2.0	1.3.E-04
GO:0050801	ion homeostasis	2.0	5.1.E-03
GO:0019725	cellular homeostasis	1.8	7.1.E-03
GO:0005694	chromosome	1.8	4.2.E-03
GO:0048878	chemical homeostasis	1.8	7.4.E-03
GO:0055114	oxidation reduction	1.6	2.5.E-03
GO:0044430	cytoskeletal part	1.6	1.2.E-03
GO:0043228	non-membrane-bounded organelle	1.5	2.7.E-06
GO:0043232	intracellular non-membrane-bounded organelle	1.5	2.7.E-06
GO:0005783	endoplasmic reticulum	1.5	9.2.E-03
GO:0005856	cytoskeleton	1.4	7.0.E-03
GO:0070013	intracellular organelle lumen	1.4	8.5.E-03
GO:0043233	organelle lumen	1.4	9.0.E-03
GO:0030554	adenyl nucleotide binding	1.3	6.9.E-03
GO:0001882	nucleoside binding	1.3	6.8.E-03
GO:0001883	purine nucleoside binding	1.3	8.6.E-03
GO:0017076	purine nucleotide binding	1.3	6.6.E-03
mmu00900	Terpenoid backbone biosynthesis	11.1	1.2.E-04
mmu00100	Steroid biosynthesis	9.1	3.4.E-04
mmu00640	Propanoate metabolism	6.0	8.3.E-04
mmu03020	RNA polymerase	5.7	3.3.E-03
mmu00620	Pyruvate metabolism	4.4	4.4.E-03
mmu00010	Glycolysis / Gluconeogenesis	3.8	1.1.E-03

Supplemental Table S8. Oligonucleotide primers used in this study

Gene symbol	5' Primer	3' Primer
For genotyping PCR		
Hsp70.3prom-Sry	aaaggcgcagggcggcgagcaggccac	gccctccatgctctctagacaattcac
Ube1	tggtctggacccaaacgctgtccaca	ggcagcagccatcacataatccagatg
For qRT-PCR		
Ddx4	gcacacgttgaatacagcggggat	tgggaggaagaacagaagaacagg
Hsd3b1	caagtgtgccagccttcatct	ttcatgattctgttcctcgtgg
Sox9	tgtgacacgggacaacacatg	ggctatccacggcacacac
Cyp19a1	cccgattcggcagcaagcgt	ccagggcccgtcagagctttc
Ldha	cactgactcctgaggaagaggccc	agctcagacgagaagggtgtggtc
Ldhb	ggacaccctgtgggacatccagaa	aagcctgggctttgatctgtgagc
Mct1/Slc16a1	ccgatgtcgacgagaagccaaagc	gctctctccaggcttcacaggtca
Mct4/Slc16a3	tggttctgggcagtggtctgttca	cagcaggcagacctggaagagcta
Acat2	gtgtctgcggcaatagctaaagaa	cagccagatgctcccagaggatg
Hmgcs1	aatgaccacagtttggatgaagga	agggagtcttggcactttcttagc
Hmgcr	agccttggcagcaggacatcttgt	tcttggtgcacgttccttgaagat
Mvk	caagtaacggcagcacacggactg	tggcttgctctagacctggcttca
Pmvk	agtagtggcctcggagcagagtcg	aaagttcccaaagttgtccagacc
Mvd	gggtccagtacatcattgccactc	gcagtccatcctggcctagcagat
Idi1	cttgaaagccgagttgggaatac	ccatcagattgggccttgtagtaa
Fdps	ttcagtgtctgctacgagcctctc	ctttcacccgagccactttttctg
Fdft1	aacatgcctgccgtcaaagctatc	gagatgacctgcttggttttgctt
Sqle	aaacttggtggagagtgtgtgacc	caacggaaaagaagtgtcgaatca
Lss	gggatcagatgtctgctagggaag	gtagctgatggcacaggacttgtt
Cyp51	cttacaggataacccagcatcagg	taggcaaaattttctccaacacaa
Tm7sf2	gggagatctcatcatggctctgg	accagcagtgcagtgaagtagagg
Msmo1	accatacgtttgctggaaaccatc	agcgcccgtataaaaaggaaccaa
Nsdhl	gacacatcttagccgctgagcac	cagaaagggattggttcatcgtt
Hsd17b7	ccacctcgggatttgggactaat	cttttccagctccagtaagacctca
Ebp	cttccgctttgtcctacagcttg	tggagtccttcgtgtagctctgtc
Sc5d	tactggattcataggggcctgcac	ggtgaaaagcatgacttgcaaacg
Dhcr7	ggtggtacctaggctggggagattg	ggagagctgcacagggtggtaca
Dhcr24	atgaggcagctggagaagtttgt	atctcccagaattcctcgcggttc
Srebf2	agctgctggagcatagcctacgg	gatggcagtagctcgctctcgtt
Ad4BP/Sf-1	aagccactctgtaggaccaagc	tgtaaatctgacgcgaaagcag
Dmrt1	gaccagtgagaagagcgggcaaac	atttggatttggggtgtggggtgc
Amh	gaacctctgccctactcggg	aagtccacggttagcaccaaa
Dhh	agcgcttccgggacctcgta	cccgctctttgcaacgctct
Scarb1	gcccacgcatgtgcaaaaacaact	aggggctgacagcagctagagttc
Abca1	gctggcaatgagtgtgccagagtt	caagacagccacaacagcagctca
Utx/Kdm6a	catcaagaaaataacaacttctgtt	aaaacaccccagtagccttcag
Uty	tgctttaatggaaaagttcattgc	gcgtaagtctcccaacacacacca
Smcx/Kdn5c	acccaaccttgtgcagtgta	gctgtagtctctttgcccgt
Smcy/Kdm5d	acagcttcctctgcccttaatccc	tgggaaacgcatacagggaatact
Rn18s	ccattcgaacgtctgccctat	gtcacccgtggtcaccatg
